# Long-Term Benefits of Chronic 5-HT2A Antagonist and 5-HT1A Agonist Treatment on ADHD Behavior in Juvenile Spontaneously Hypertensive Rats

**DOI:** 10.2174/011570159X409741251202071559

**Published:** 2026-02-20

**Authors:** Sampath Madhyastha, Muddanna S. Rao

**Affiliations:** 1 Department of Anatomy, College of Medicine, Kuwait University, Kuwait City, Kuwait

**Keywords:** ADHD, dopamine receptor, prefrontal cortex, SHR, Striatum, 5-HT2A receptor, 5-HT1A receptor

## Abstract

**Introduction:**

Norepinephrine and Dopamine (DA) are critical brain amines in Attention Deficit Hyperactivity Disorder (ADHD), with treatments targeting their neuroreceptors often causing adverse effects, particularly in the motor system through DA receptors. Our earlier study suggested that chronic treatment with a 5-HT1A receptor agonist or a 5-HT2A receptor antagonist in juvenile spontaneously hypertensive rats (SHRs) may improve ADHD-like symptoms by potentially modulating DA receptors. This study investigated the long-term impacts of these serotonergic receptor manipulations on ADHD behavior.

***Methods*** **:** Male SHRs (15 days old) received either ipsapirone (a 5-HT1A agonist) or MDL100907 (a 5-HT2A antagonist) from postnatal day (PND) 15 to 42, in parallel with similarly aged Wistar Kyoto rats (WKY). After eight weeks of undisturbed observation, we assessed hyperlocomotor activity, anxiety, and impulsivity. On PND 122, rats were sacrificed, and the striatum and prefrontal cortex (PFC) were analyzed for DA-D1, DA-D2, 5-HT1A, and 5-HT2A receptor proteins and DA levels.

**Results:**

The results showed that both treatments had lasting positive effects on ADHD behaviors, linked to increased 5-HT1A and 5-HT2A receptors in the PFC and striatum. Ipsapirone (5-HT1A agonist) did not alter DA receptor expression but reduced DA levels, while the 5-HT2A antagonist reduced DA-D2 and increased DA-D1 expression, with enhanced DA content levels. In the striatum, both treatments increased DA-D2 and reduced DA-D1 receptors, but the 5-HT1A agonist lowered DA content.

**Discussion:**

Striatal DA-D2 dysregulation appears to contribute to hyperlocomotor activity, while attentional impairments are associated with enhanced striatal DA-D2 receptor expression. Enhancing 5-HT activity either through 5-HT2A antagonists or 5-HT1A agonists effectively alleviates core ADHD symptoms in SHRs, likely by indirectly modulating DA signaling. These serotonergic agents may influence DA pathway activity *via* cortical mechanisms, providing therapeutic benefits without the long-term adverse effects typically associated with direct DA receptor blockade.

**Conclusion:**

These findings suggest that dysregulation of 5-HT1A and DA-D2 receptors may contribute to ADHD, and monotherapy with either a 5-HT1A receptor stimulator or a 5-HT2A receptor inhibitor can effectively alleviate core ADHD symptoms long-term, likely through indirect DA system modulation.

## 1 INTRODUCTION

 Attention Deficit Hyperactivity Disorder (ADHD) is a neurobehavioral condition characterized by persistent patterns of inattention, excessive activity, and impulsive behavior that disrupt daily functioning. It often begins in childhood and affects various areas of life, including social relationships, academic performance, and work. Although commonly diagnosed in children and adolescents, ADHD also impacts many adults. The worldwide prevalence of ADHD is estimated at 7.6% in children (ages 3-12) and 8.0% in adolescents [1, 2]. Approximately 7 million children in the United States (11.4%) aged 3-17 have been diagnosed with ADHD, based on a 2022 national survey [3].

Reduced dopamine (DA) and noradrenaline (NE) activity are linked to ADHD, as supported by PET scan findings. The disorder also involves functional and structural changes in the frontal and prefrontal cortex, as well as other brain regions such as the cerebellum, parietal lobe, and basal nuclei [4-7]. ADHD involves two major brain networks:

a) **Fronto-striatal network** - connects the frontal lobe and striatum. Dysfunction in this network leads to inattention, hyperactivity, impulsivity, and cognitive impairment [8]. It receives input from dopaminergic, serotonergic, noradrenergic, and cholinergic neurons.

b) **Mesocortical pathway** - a dopaminergic pathway connecting the midbrain tegmental area to the prefrontal cortex (PFC). DA input through this pathway inhibits PFC neurons, and dysfunction contributes to ADHD-related behavioral problems [9].

Both DA and NE influence cognitive and executive functions, which are often impaired in ADHD [10]. Research highlights a key role for NE in ADHD and supports the therapeutic importance of NE-targeting medications [11], while low NE levels, particularly in the prefrontal cortex are linked to impaired working memory and attention [12]. DA receptors D1 and D2 are located in brain regions controlling reward, memory, learning, and movement [13]. Individuals with ADHD often have increased DA transporters (DAT), which reabsorb DA and potentially alter DA levels [14]. NE plays a critical role in ADHD, particularly in the PFC, where low NE levels impair attention and working memory [12]. Research supports the efficacy of NE-targeting medications in managing ADHD symptoms [11].

Studies show that stimulant medications, such as Methylphenidate and Amphetamine, enhance DA and NE activity within the prefrontal cortex (PFC), providing insight into ADHD pathophysiology [15]. However, these drugs are contraindicated in some children due to side effects, including increased heart rate, elevated blood pressure, appetite loss, sleep disturbances, irritability, abuse potential, and risks of stroke, heart attack, or sudden death [16].

Non-stimulant medications, such as Atomoxetine (a NE reuptake inhibitor) and Clonidine or Guanfacine (alpha-2 adrenergic agonists), are also used in ADHD management [17]. Alpha-2 agonists act on presynaptic receptors to regulate NE release but may cause side effects such as fatigue, irritability, headache, weight gain, and increased suicide risk [18]. Both stimulant and non-stimulant treatments have limitations and risks [19].

Although atypical antipsychotics are not FDA-approved for ADHD, evidence supports their use in treating severe symptoms. These medications target DA receptors, raising concerns due to the distinct roles of DA receptors in different brain regions. For example, mesolimbic dopaminergic pathways are overactive, stimulating DA-D2-like receptors, while mesocortical pathways to the PFC are underactive, affecting D1 receptors and contributing to adverse symptoms [20]. Most DA agonists target D1, D2, or D3 receptors, which can lead to side effects such as addiction-related behaviors, including gambling, compulsive shopping, and hypersexuality [21]. Chronic use may result in movement disorders, cardiovascular complications, and psychiatric problems [22, 23].

Molindone, a DA antagonist, has shown benefits in reducing ADHD symptoms but can also cause adverse effects, including somnolence, akathisia, and weight gain [24]. The use of DA agonists in children is particularly concerning due to rapid metabolism, which can cause hypotension and other side effects. These limitations highlight the need for safer and more effective treatment strategies.

Recent research highlights the role of the serotonin system in modulating DA activity to reduce core ADHD symptoms [25]. Elevating 5-HT levels restores the balance between DA and 5-HT, helping to minimize hyperactivity [26]. Serotonin receptors, specifically 5-HT1A and 5-HT2A, are closely associated with ADHD-related behaviors [27, 28]. Although atypical antipsychotics are not FDA-approved for ADHD, risperidone, an antagonist of 5-H2A, D2 and alpha1 and alpha 2 adrenergic receptor has been shown to improve attention and reduce hyperactivity [29]. Quetiapine increases prefrontal DA release by inhibiting 5-HT2A receptors while partially stimulating 5-HT1A receptors, thereby alleviating ADHD symptoms and aggression [30].

In clinical practice, stimulants or non-stimulants are sometimes combined with atypical antipsychotics depending on ADHD severity. These findings suggest that blocking 5-HT2A and stimulating 5-HT1A receptors can significantly reduce ADHD symptoms without directly manipulating NE or DA receptors. This pharmacological approach allows atypical antipsychotic medications to influence dopaminergic function without extensive blockade of D2 receptors.

In our earlier study, we observed that serotonergic manipulation through 5-HT2A blockade and 5-HT1A stimulation suppressed ADHD-like behaviors in juvenile spontaneously hypertensive rats (SHRs) [31]. However, the prolonged impact of altering serotonergic receptors on ADHD behavior remains unexplored. A further challenge in children with ADHD is the significant developmental changes in neuroreceptor expression across different brain regions. These changes may reflect adaptive processes in the developing brain, suggesting that assessing neuroreceptor expression during early adulthood would be an ideal approach.

## 2 MATERIALS AND METHODS

### 2.1 Subjects

This study involved fifteen-day-old male Spontaneously Hypertensive Rats (SHRs) and Wistar-Kyoto (WKY) rat pups, which were bred and maintained at the Animal Resources Center, Health Sciences Center (HSC), Kuwait University. The SHR strain was originally obtained from Charles River Laboratories (Wilmington, MA, USA) and has since been sustained at the HSC facility. All animals were housed in sterile polypropylene cages under standardized environmental conditions (temperature: 22 ± 2°C; light/dark cycle: 12 hours each). Until 21 days of age, the pups remained with their mothers before being weaned and had free access to a normal diet and water.

Animal care and drug treatment protocols adhered to guidelines approved by the Institutional Animal Care and Use Committee of Kuwait University, in compliance with the NIH Guidelines and the Guide for the Care and Use of Laboratory Animals (Approval No. 23/VDR/EC/3560, dated 7 November 2019). All experiments were conducted in accordance with the standards set forth in the US National Research Council's *Guide for the Care and Use of Laboratory Animals*, and we endorse the ARRIVE guidelines.

### 2.2 Study Design

This study investigates whether therapeutic intervention in Spontaneously Hypertensive Rats (SHRs), a well-established model of ADHD, using a serotonergic receptor modulator, can effectively alleviate ADHD symptoms over an extended period. The study employs a validated animal model with an adequate number of subjects in each group to meet statistical requirements. All experimental parameters are well-established and widely cited in the literature.

SHR and Wistar-Kyoto (WKY) rat pups aged fifteen days were used and assigned to six different groups (n = 12 per group):

- Normal Control (SHR-NC),
- Wistar-Kyoto Normal Control (WKY-NC),
- SHR-MDL (MDL 100907/5-HT2A antagonist),
- WKY-MDL (5-HT2A antagonist),
- SHR-IPS (ipsapirone/5-HT1A agonist),
- WKY-IPS (ipsapirone/5-HT1A agonist).

From postnatal day (PND) 15 to 42, rat pups in each treatment group received a four-week regimen of receptor agonists or antagonists. As treatment began at PND 15, pups remained with their dams until weaning on PND 21. Post-weaning, rats were grouped into cages containing 2-3 animals each. Untreated SHR pups of the same age (SHR-NC group) served as the ADHD model, while similarly aged WKY pups were designated as the control group (WKY-NC group). The study period (PND 15-43) corresponds to the developmental stages of childhood to early adolescence in humans [32].

Following treatment, the rats remained undisturbed for eight weeks (PND 43-99). After this period, all groups underwent a series of behavioral tests over eight weeks (PND 100-121), including the open field test to measure locomotor activity, a modified T-maze to assess impulsivity, evaluation of impulsive water consumption in an aversive electro-foot shock setup, and the elevated plus maze.

On PND 122, following completion of behavioral assessments, rats were either perfused with 4% paraformaldehyde for immunohistochemical detection of 5-HT1A, 5-HT2A, DA-D1, and DA-D2 receptors in the PFC and striatum (n = 6 per group) or their fresh brain tissues were harvested for biochemical quantification of these receptors and dopamine concentrations in the same brain regions (n = 6 per group).

### 2.3 Drug Preparation and Dosage

The 5-HT1A agonist ipsapirone (IPS, Cat. No. 1869) and the 5-HT2A antagonist MDL 100907 (MDL, Cat. No. 4173) were procured from Tocris Bioscience, United Kingdom. Stock solutions were initially prepared using DMSO and stored at -20°C at concentrations of 4 mg/mL for IPS and 2 mg/mL for MDL. Working injection solutions were freshly prepared daily by diluting the stock solutions with saline to achieve final concentrations of 200 µg/mL for IPS and 100 µg/mL for MDL.

Drugs were administered intraperitoneally at the following doses:

- **MDL:** 0.5 mg/kg (1^st^ week), 0.75 mg/kg (2^nd^ week), 1 mg/kg (3^rd^ and 4^th^ weeks)
- **IPS:** 1 mg/kg (1^st^ week), 1.5 mg/kg (2^nd^ week), 2 mg/kg (3^rd^ and 4^th^ weeks)

Dosage selection was guided by previous studies [33]. The amount of drug administered to each rat was calculated based on its body weight at the beginning of each week. Control groups received a matching volume of saline containing DMSO (50 µL DMSO per mL of saline).

### 2.4 Behavioral Tests (for Assessment of ADHD)

#### 2.4.1 Assessment of General Locomotion and Exploratory Activity in a Novel Setting

To evaluate spontaneous movement and exploratory behavior, the open field test was employed. This behavioral assay is commonly used to assess both locomotor activity and anxiety-like behavior in rodents. The testing apparatus consisted of a transparent Plexiglas enclosure measuring 90 cm × 90 cm with a height of 20 cm. The arena was placed beneath a camera connected to the EzVideo™ tracking system (version 5.70, Accuscan Instruments, Columbus, OH, USA).

For data analysis, the floor was virtually divided into central and peripheral regions. Each animal was placed in the center of the arena and allowed to explore freely for 10 minutes, during which its movement patterns were recorded. The tracking software quantified parameters such as total distance traveled and the time spent in each zone. Greater total distance indicated hyperactivity, while a preference for the periphery over the center was interpreted as increased anxiety [34].

#### 2.4.2 Evaluation of Locomotor Hyperactivity

To measure sustained hyperactivity in a non-novel environment, all animals underwent a locomotor activity assessment lasting 60 minutes, as previously described [34]. The test was conducted in a square black Plexiglas chamber (60 × 60 × 60 cm) placed beneath a video-based tracking system. To eliminate the confounding effects of novelty, data from the first 15 minutes of the session were excluded. Rats’ activity was continuously recorded for the remaining 45 minutes. The tracking system captured behavioral metrics, including total movement time (the duration the animal was active), frequency of movement episodes (defined as one or more position changes exceeding a minimum threshold within three seconds), resting time (periods of immobility), and total distance traveled. All parameters were processed and analyzed using the accompanying video tracking software.

#### 2.4.3 Assessment of Anxiety-Like Behavior Using the Elevated Plus Maze

To evaluate anxiety-related responses, rats from all experimental groups were tested using the elevated plus maze (EPM). This apparatus featured a cross-shaped layout with four arms radiating from a central platform. Each arm measured 30 cm in length and 10 cm in width and was elevated 30 cm above the ground. Two arms were open and lacked walls, while the remaining two were enclosed with 30 cm-high side walls. The maze was placed on a table beneath a camera connected to a video-tracking system. At the start of each trial, a rat was placed in the center of the maze, where all four arms intersected, and allowed to move freely for 8 minutes. The tracking system recorded the amount of time the animal spent in both the open and closed arms. These data were used to calculate the percentage of time spent in each arm type using a predefined formula [35].


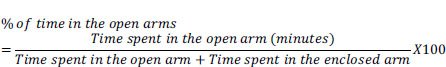


#### 2.4.4 Assessment of Impulsivity in the Modified T-Maze Task

Impulsive behavior was evaluated through the use of a modified T-maze setup. The maze consisted of one starting arm and two goal arms, each measuring 30 cm in length, 10 cm in width, and 30 cm in height, and a pair of goal arms with the same measurements. Each arm featured a sliding door at its entrance, and a goal area (goal end) featured a food well at its end, partitioned from the stem by a separate sliding barrier [36]. Prior to testing, rats were food-deprived for 30-36 hours, and thereafter, their food intake was limited to 5-6 pellets (4-5 g each) per day, given after the daily session. This restriction maintained their body weight at 80% of their pre-deprivation weight. The evaluation of impulsive behavior was divided into three separate stages:

##### 2.4.4.1 Acclimatization Phase

This phase took place on the first day and included two sessions, spaced 2 hours apart, each consisting of 5 trials. During each trial, three food pellets were positioned in the goal zones of both arms. The rat was placed on the start arm and allowed to explore both goal arms freely and to consume the pellets. Once the rat had eaten the pellets from both sides, or after a maximum of 3 minutes, it was returned to the start arm to begin the next trial.

##### 2.4.4.2 Training Phase

This phase was carried out from Day 2 to Day 4, with rats undergoing three training sessions per day. Each session included five trials and was spaced two hours apart. In every trial, the right goal arm was baited with three food pellets, representing a high-reward option, while the left goal arm contained a single pellet, serving as a low-reward alternative. Each trial commenced with the rat placed in the start arm, where it was allowed to navigate the maze and choose between the arm containing the larger reward and the one containing the smaller reward. Upon selecting an arm and consuming the food pellets within it, the trial was concluded, restricting access to the alternate arm for that session. Following a one-minute break, the subsequent trial began by repositioning the rat in the start arm. The selected arm was documented for every trial, and the proportion of choices made for each arm per session was computed using the following formula:

Percentage of selections for the large reward arm = (total selections of the large reward arm ÷ 5) × 100

Percentage of selections for the small reward arm = (total selections of the small reward arm ÷ 5) × 100

Training sessions were conducted over three consecutive days, continuing until each rat reliably selected the large reward arm in at least 80% of trials across two consecutive sessions—equivalent to four out of five correct choices per session. Most animals met this criterion within 7 to 9 sessions. Upon reaching this performance benchmark, the testing phase was initiated and spanned five additional sessions over the following two days. The reward structure remained unchanged from the training phase, with three pellets placed in the large reward arm and one in the small reward arm. During each test trial, if a rat chose the small-reward arm, it was allowed to proceed directly to the goal area, consume the pellet, and the trial was terminated. In contrast, selecting the large-reward arm resulted in a 30-second delay, during which the rat was confined by sliding doors at the arm's entrance and near the goal zone. After the delay, the rat accessed and consumed the reward, marking the end of the trial. The chosen arm for each trial was recorded, and the proportion of entries into each arm was computed using the following method:

Percentage of selections for the delayed large reward arm = (total number of entries into the delayed large reward arm ÷ 5) × 100

Percentage of selections for the small reward arm = (total number of entries into the small reward arm ÷ 5) × 100

A reduction in the frequency of choosing the delayed large reward arm was considered reflective of increased impulsivity.

#### 2.4.5 Analysis of Impulsive Responses in the EFSDT (Electro Foot Shock Aversive Drinking Test)

Impulsive behavior was evaluated using the EFSDT, as previously described [35]. The testing chamber was constructed from black Plexiglas and had dimensions of 40 × 40 × 40 cm. The base of the apparatus featured a metal grid floor connected to a shock delivery system. This floor was divided into four functional zones: a Start Box (10 × 10 cm), a central Choice Zone (40 × 20 cm), a Water Access Zone (20 × 20 cm), and a Dummy Water Zone (20 × 20 cm). A Perspex divider separated the actual water zone from the dummy water zone. The start area was connected to the choice zone *via* a sliding door, allowing the animal to enter and select between the two forward zones. Both the water and dummy areas were equipped with stainless-steel drinking spouts extending 4 cm into the chamber from the outer wall, each positioned 6 cm above the floor. During the experimental sessions, only the water bottle in the designated water zone was filled, while the dummy zone remained empty. The entire setup was positioned beneath the EzVideo™ video tracking system to monitor and record the animals' behavior. Prior to testing, the rats underwent a water-deprivation period lasting 30-36 hours. After each daily session, access to water was allowed for a duration of 15 minutes. The experimental protocol was divided into three key stages: (i) acclimatization, (ii) training, and (iii) evaluation.

##### 2.4.5.1 Acclimatization Phase

This stage involved a single session comprising five individual trials conducted on the first day. For each trial, the rat was placed in the start compartment, after which the sliding door was opened, allowing the animal to freely explore both the water and dummy zones for 3 minutes. No water was provided in either area during this initial phase.

##### 2.4.5.2 Training Phase

The training phase consisted of four sessions (two per day, spaced 2 hours apart), each with 5 trials and a 1-minute break between trials. In every trial, the rat was placed in the start area and allowed to explore both the water and dummy zones. A trial concluded when the rat drank from the water zone for 5 seconds or stayed in the dummy zone for over 3 seconds. Water zone entries were tracked, and entry percentages were calculated per session. The testing phase began once a rat consistently chose the water zone in at least 80% of trials (4 out of 5), a criterion usually reached within 3 to 4 sessions.

##### 2.4.5.3 Evaluation Phase

After meeting the training criteria, the impulsivity evaluation phase was initiated. This stage included two sessions, each comprising five trials. The procedure mirrored the training phase, with one key difference: when a rat attempted to drink water, it received a mild foot shock (2 mA for 0.5 seconds), signaling the end of the trial. Entry into the water zone was tracked *via* video recording, and the percentage of entries was calculated per session. Additionally, manual counts were taken for each drinking attempt. A higher frequency of entries into the water area and more drinking attempts were interpreted as signs of increased impulsivity [35].

### 2.5 Assessment of Protein Expression through Immunoblotting

For protein expression analysis *via* western blotting, rats were euthanized through carbon dioxide inhalation and subsequently perfused with 100 mL of ice-cold saline. Brains were quickly extracted on a chilled dissection surface. Target brain areas-including the prefrontal cortex (PFC), dorsal striatum, and substantia nigra-were precisely isolated in cold saline, weighed, and instantly frozen in liquid nitrogen. These tissue samples were preserved at -80°C until used for analysis. Prior to processing, the frozen tissues were thawed at 4°C and homogenized in a measured volume of RIPA buffer. The buffer composition included 50 mM Tris, 1% NP-40, 5 mM EDTA, 0.1% SDS, 0.5% sodium deoxycholate, and protease inhibitors such as PMSF (10 µL/mL) and benzamidine (2 mM), along with 100 µL/mL of a protease inhibitor cocktail (Sigma Chemicals). The homogenized samples were centrifuged at 14,000×g for 20 minutes at 4°C to remove debris. Protein concentration was then quantified using the Bradford assay. To denature the proteins, the samples were heated in a water bath at boiling temperature for 5 minutes. Subsequently, 30 µg of protein per sample was loaded into each well and separated using electrophoresis on a 10% SDS-polyacrylamide gel. Following separation, proteins were transferred onto a PVDF membrane (Millipore, Bedford, MA, USA) using a blotting system (Bio-Rad Laboratories, Hercules, CA, USA). To prevent non-specific antibody binding, the membrane was incubated for 2 hours at room temperature in a blocking solution containing 5% skim milk and 0.05% Tween-20 in tris-buffered saline (TBST). The membrane was then incubated overnight at 4°C on a shaker with appropriately diluted primary antibodies targeting specific receptors. These included rabbit polyclonal antibodies against the 5-HT1A receptor (Abcam-85615), 5-HT2A receptor (Abcam-66049), dopamine D1 receptor (DA-D1, Abcam-20066), and dopamine D2 receptor (DA-D2, Abcam-21218). After primary antibody incubation, the membrane was rinsed three times using TBST and subsequently incubated with horseradish peroxidase (HRP)-linked secondary antibodies (goat anti-rabbit IgG-HRP, Abcam-205718) for 2 hours at room temperature. This step was followed by additional washes with TBST to remove unbound antibodies. To estimate the target proteins, the PVDF membrane was treated with an enhanced chemiluminescence (ECL) reagent and subsequently exposed to X-ray film. This method was used to detect bands corresponding to 5-HT1A, 5-HT2A, and dopamine receptors D1 and D2 [37]. To confirm consistent protein loading across all lanes, the membrane was stripped and re-incubated with a β-actin-specific antibody (Abcam, Cambridge, UK; dilution 1:1000). The β-actin signal was also visualized using ECL detection. Band intensities were quantified using a GS-800 calibrated densitometer, and results were normalized against β-actin expression to ensure accuracy and comparability [38].

### 2.6 Tissue Immunolabeling

For immunolabeling, rats were euthanized *via* CO_2_ inhalation and underwent transcardial perfusion with 100 mL of phosphate-buffered saline (PBS), followed by perfusion with freshly prepared 4% paraformaldehyde in 100 mM phosphate buffer (pH 7.4). Following this procedure, the brains were carefully removed and immersed in the same fixative for 48 hours of post-fixation. To achieve cryoprotection, the brains were sequentially transferred through increasing concentrations of sucrose solutions (10%, 20%, and 30%), spending 24 hours in each. Coronal brain sections (30 μm thick) were then sliced using a cryostat (Leica, Nußloch, Germany) and placed into 24-well plates containing phosphate buffer. Selected free-floating sections corresponding to the PFC, dorsal striatum (caudate putamen), and substantia nigra of the midbrain were used for immunostaining. These sections were processed to detect 5-HT1A, 5-HT2A, DA-D1, and DA-D2 receptor expression. To suppress endogenous peroxidase activity, tissue sections were incubated in 3% hydrogen peroxide (H_2_O_2_) for 30 minutes. This was followed by a 30-minute incubation in blocking serum containing 0.3% Triton X-100, which served to reduce non-specific binding of primary antibodies. After blocking, the sections were incubated overnight at 4°C with diluted primary antibodies-the same antibodies utilized in the Western blot experiments. The following day, sections were incubated with a biotin-conjugated goat anti-rabbit secondary antibody for 1 hour. Subsequently, they were incubated for another hour with the avidin-biotin-peroxidase complex (ABC). Immunoreactive signals were visualized using 3,3′-diaminobenzidine (DAB) as the chromogen. Between each step, sections were rinsed three times in PBS to remove unbound reagents. For negative controls, some sections were processed without primary antibodies to confirm staining specificity. After immunostaining, brain sections were placed on gelatin-coated slides and allowed to air-dry overnight. The next day, sections were counterstained using hematoxylin to enhance tissue contrast. After staining, the samples were dehydrated through a series of ethanol concentrations, cleared using xylene, and then sealed with coverslips using Permount mounting medium (Fisher Scientific, Pittsburgh, PA, USA). High-resolution photomicrographs were obtained using an Olympus DP75 digital camera attached to an Olympus microscope, utilizing a 40× objective lens. For the PFC, analyses were conducted in the medial, lateral, and orbital subregions. In the case of the striatum, only the dorsal region was included in the evaluation of immunostaining for 5-HT1A [39], 5-HT2A [40], and dopamine D1 and D2 receptors [41].

### 2.7 ELISA for Dopamine Estimation

A microplate pre-coated with a monoclonal antibody targeting DA serves as the platform for a competitive binding assay. In this setup, biotin-tagged DA competes with untagged DA in the sample for binding to the immobilized DA-specific antibody [42]. Brain homogenates prepared from the PFC and striatum were subjected to centrifugation at 3000 rpm for 3 minutes. The collected supernatant was subsequently analyzed to quantify DA concentrations in both regions using a commercially available ELISA kit (MBS2700357). Wells were prepared for diluted standards, blanks, and samples. Five wells were allocated for preparing the standard curve, and one well was reserved as a blank control. To each designated well, 50 μL of the corresponding standard solution, blank, or test sample was added, followed immediately by 50 μL of Detection Reagent A.

The plate was gently agitated on a microplate shaker, covered with a plate seal, and incubated at 37°C for 60 minutes. The appearance of detection reagent A was cloudy. Following the incubation period, the plate was brought to room temperature and gently agitated to ensure homogeneity of the contents. The liquid was then aspirated from each well, and 350 μL of 1X wash buffer was dispensed into each well using an automated plate washer. The plate was then allowed to settle for 1-2 minutes. Any leftover liquid in the wells was eliminated by repeatedly tapping the plate onto absorbent paper. The plate was turned upside down and gently pressed against absorbent paper to completely remove any remaining liquid. Next, 100 μL of the prepared Detection Reagent B solution was dispensed into each well. The plate was then covered and incubated at 37°C for half an hour. The aspiration and washing steps were carried out five times. Afterward, 90 μL of substrate solution was introduced into each well, and the plate was incubated at 37°C for 10 to 20 minutes in the dark to prevent light exposure. As a result, a blue coloration developed in the wells. This reaction was halted by adding 50 μL of the stop solution to each well, which turned the solution yellow. The plate was gently tapped to ensure proper mixing of the contents, then absorbance was measured at 450 nm using a microplate reader.

### 2.8 Statistical Analysis

All results were presented as mean ± standard error (SE) and evaluated using SPSS statistical software (version 25). A two-way Analysis of Variance (ANOVA) was employed for statistical comparisons, followed by Bonferroni’s post hoc test for multiple comparisons. A p-value less than 0.05 was regarded as statistically significant.

## 3 RESULTS

### 3.1 General Locomotion and Exploratory Activity in a Novel Setting

**Fig. (1):**
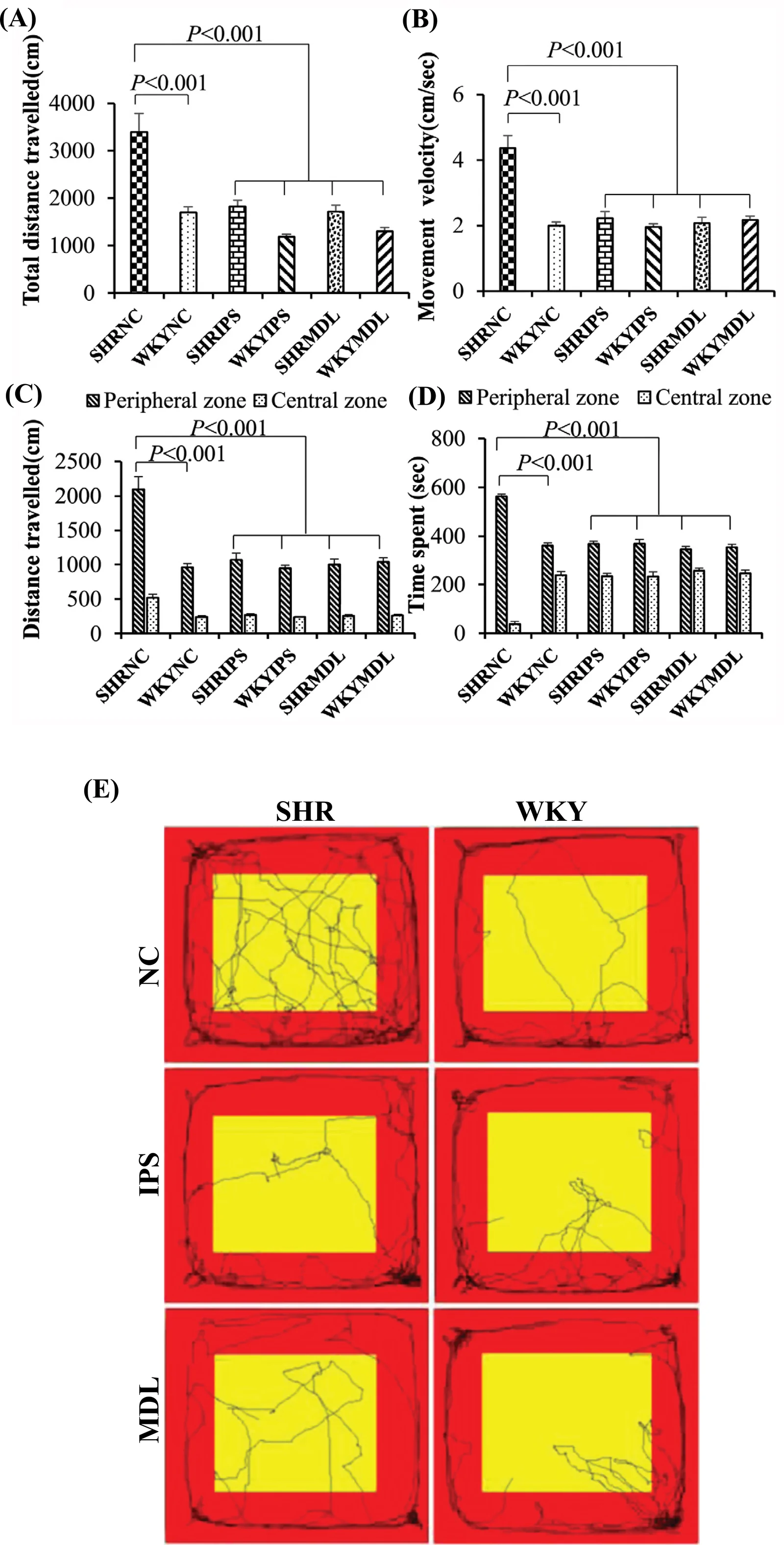
(**A**) Total distance traveled, (**B**) Movement velocity, (**C**) Distance traveled in central(yellow) and peripheral (red) zones, and (**D**) Time spent in central and peripheral zones, in the open field test apparatus during the open field test for 10 minutes. SHR-NC rats showed significantly higher distance and movement velocity than WKY-NC rats. These measures were reduced in the SHR-MDL and SHR-IPS groups compared with the SHR-NC group and were comparable to the respective WKY groups. (**E**) Sample tracking shows increased movement in SHR-NC rats. Activity in both central and peripheral zones was lower in MDL and IPS-treated SHR rats compared to SHR-NC (n=12).

Untreated SHR rats exhibited significantly greater average distance traveled and movement speed (*p <* 0.001) compared to the untreated WKY control group. Administration of MDL or IPS to SHR rats resulted in a marked decrease (*p <* 0.001) in both parameters when compared to the untreated SHR group (Figs. **1A** and **1B**). Likewise, WKY rats that received MDL or IPS exhibited reduced movement distance and speed relative to SHR controls; however, their values did not differ significantly from those of the WKY control group. Moreover, untreated SHR rats covered a significantly longer distance and spent more time in the outer area of the open field than WKY controls (*p <* 0.001). Both MDL and IPS treatments led to a significant decrease (*p <* 0.001) in these measures in SHR and WKY rats compared to the untreated SHR group (Figs. **1C** and **1D**). Illustrative video-tracking data are presented in Fig. (**1E**). The heightened anxiety-related behavior typically seen in young adult SHR rats, evidenced by increased time spent in the periphery of the open field, was mitigated following early-life administration of MDL or IPS.

### 3.2 Assessment of Locomotor Hyperactivity in the 60-Minute Open Field Test

**Fig. (2):**
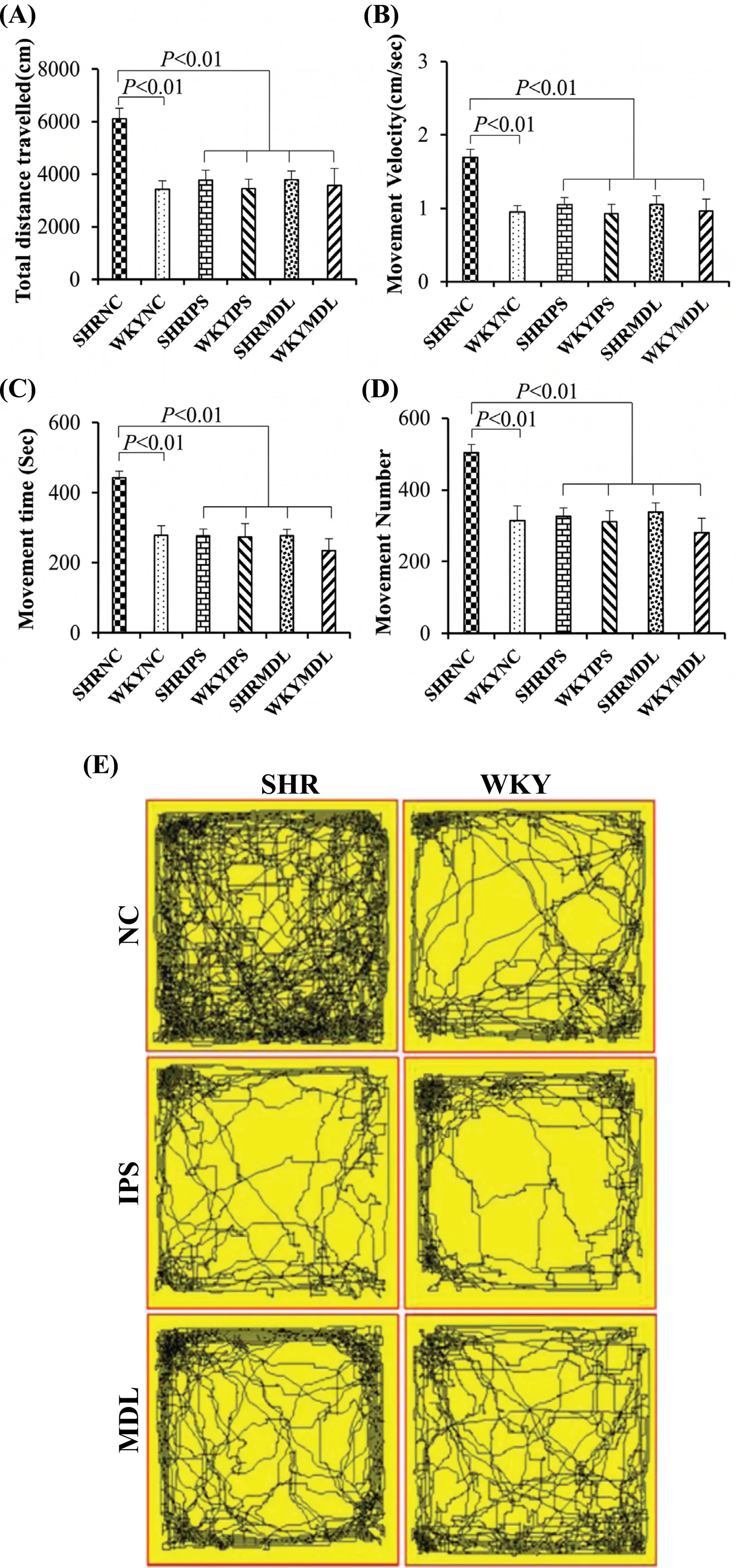
(**A**) Distance traveled, (**B**) Movement velocity, (**C**) Movement time, and (**D**) Number of movements for one hour locomotor activity test in different rat groups. SHR-NC rats showed significantly higher values in all parameters compared to WKY-NC rats. These measures were notably reduced in the SHR-IPS and SHR-MDL groups compared with the SHR-NC group and were comparable to the respective WKY groups. (**E**) Sample video tracking illustrates increased activity in SHR-NC rats, which was markedly lower in IPS- and MDL-treated SHR rats (n=12).

During the one-hour observation period, SHR control rats exhibited significantly greater average distance traveled and movement velocity (*p <* 0.01) compared to WKY control rats. Administration of MDL or IPS to SHR rats led to a significant reduction (*p <* 0.01) in these locomotor measures when compared with both untreated SHR and WKY controls (Figs. **2A** and **2B**). Additionally, WKY control rats displayed significantly fewer movements and reduced total movement duration (*p <* 0.01) than SHR controls. Similar reductions in movement count and movement number were also observed in MDL- and IPS-treated SHR rats, with values significantly lower than those of the SHR control groups (Figs. **2C** and **2D**). Sample-tracking visuals are shown in Fig. (**2E**). These findings indicate that early-life administration of MDL or IPS effectively reduced hyperactive behavior in young adult SHR rats.

### 3.3 Elevated Plus Maze Analysis of Anxiety Responses

**Fig. (3):**
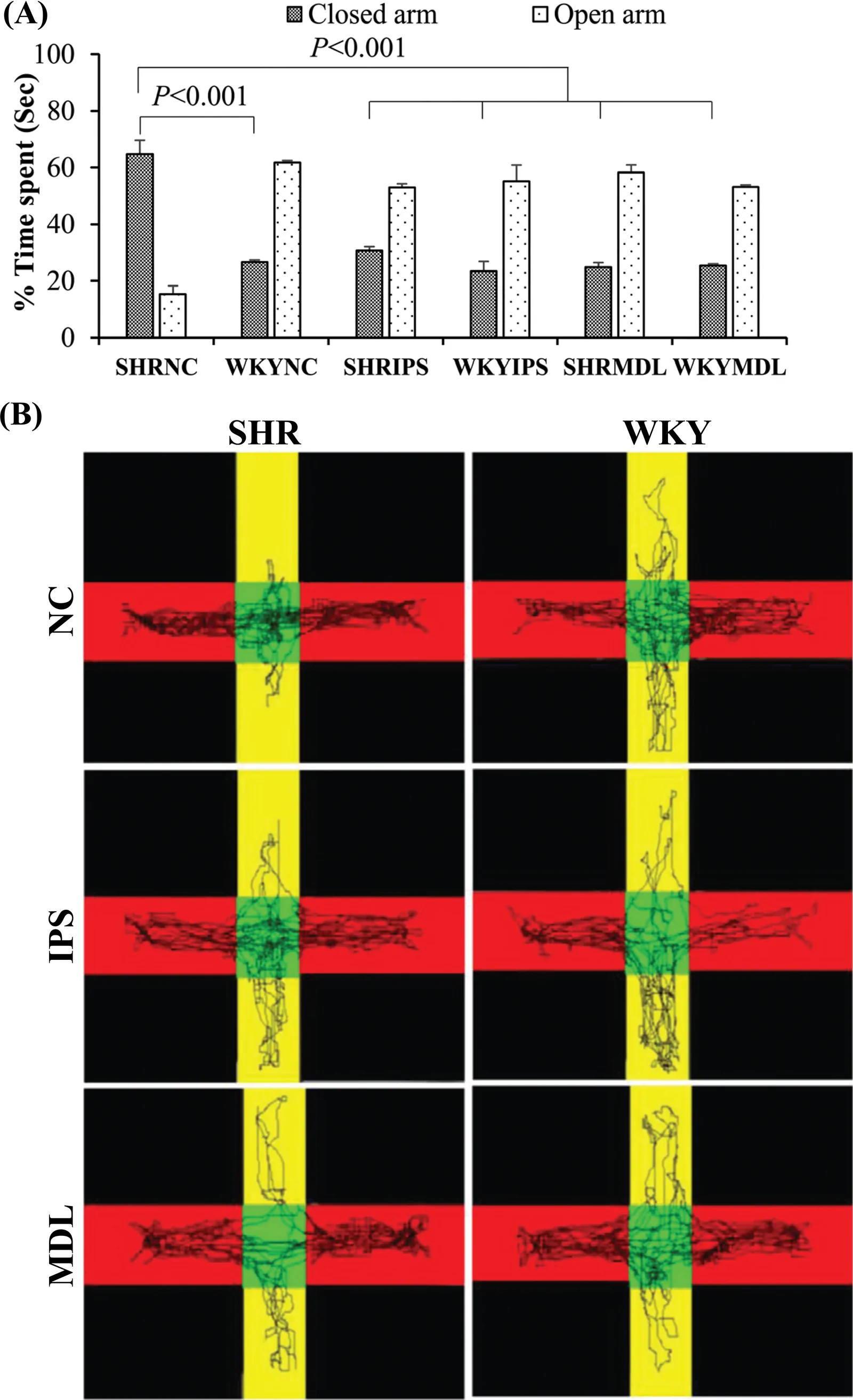
(**A**) Percentage of time spent in open and closed arms of the elevated plus maze on test day. SHR-NC rats spent significantly more time in the closed arms than WKY-NC rats. Time spent in closed arms was reduced in SHR-IPS and SHR-MDL groups, indicating less anxiety-like behavior. (**B**) Sample video tracking showing increased closed-arm(red) exploration by SHR-NC rats. In contrast, SHR-IPS and SHR-MDL rats spent more time on the open arms (yellow), similar to WKY-NC rats (n=12).

SHR control rats showed a significantly reduced percentage of time in the open arms of the elevated plus maze compared to WKY controls (*p <* 0.01). Conversely, they spent considerably more time in the closed arms (*p <* 0.001) than their WKY counterparts. On the other hand, WKY control rats exhibited a markedly lower proportion of time in the closed arms than SHR control rats. Treatment of SHR rats with MDL or IPS resulted in a significant decrease (*p <* 0.001) in the amount of time spent in the closed arms of the elevated plus maze when compared to both untreated SHR and WKY control groups (Fig. **3A**). Sample tracking data are presented in Fig. (**3B**). These findings indicate that early-life administration of MDL or IPS effectively alleviated the anxiety-like behaviors observed in young adult SHR rats.

### 3.4 Measurement of Impulse Control Through Modified T-Maze Paradigm

**Fig. (4):**
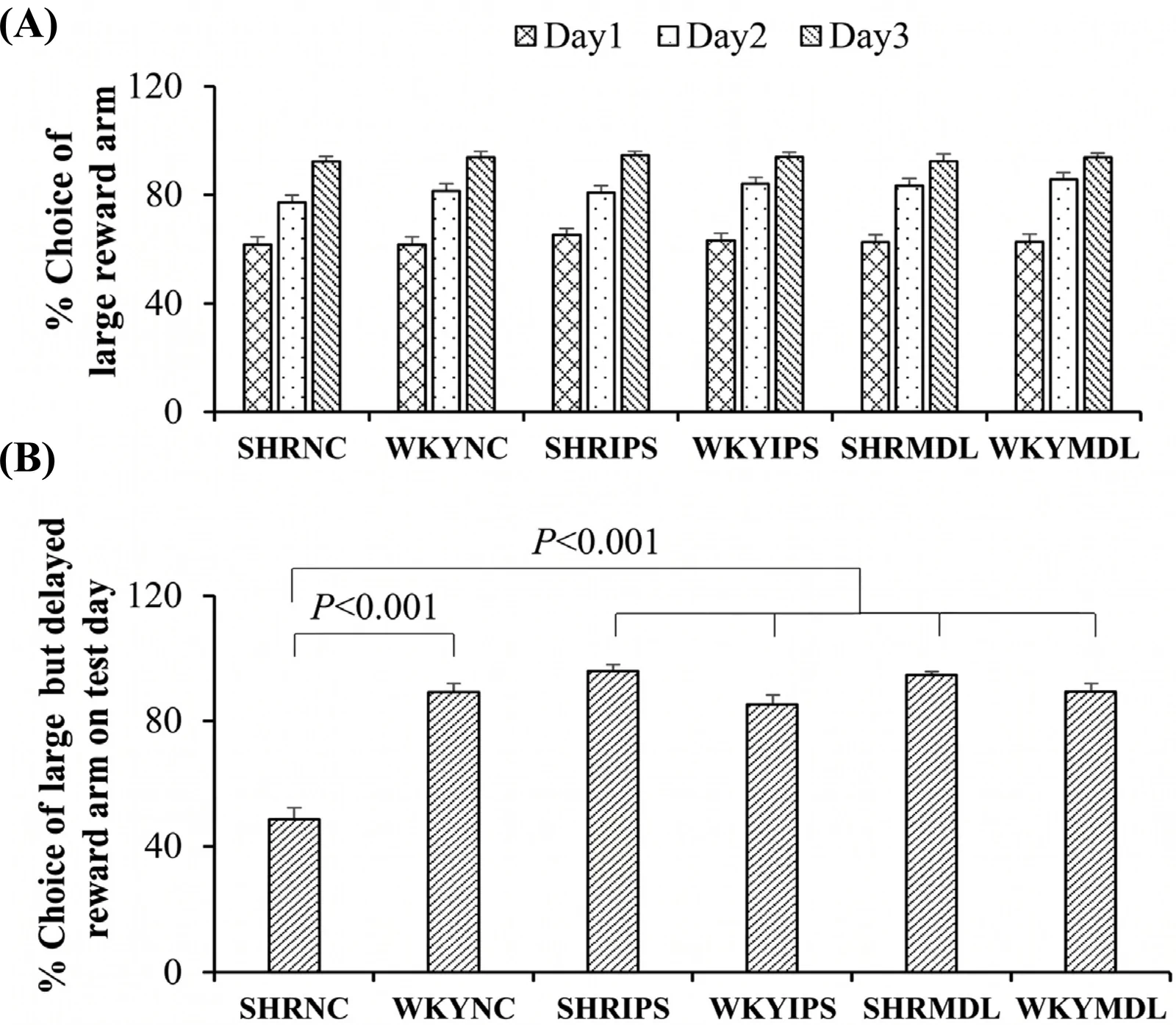
(**A**) Percentage selections of the large reward arm across the days 1-3 of the learning phase. All groups showed a steady increase in choosing the large reward arm during the learning phase. (**B**) Percentage selections of the large, delayed reward arm on the test day in the modified T-maze impulsivity task. However, during the test session, SHR-NC rats chose the large but delayed-reward arm significantly less often than WKY-NC rats. In contrast, IPS- and MDL-treated SHR rats made significantly more choices for the delayed reward, showing improved impulsive control compared to the SHR-NC group (n=12).

As anticipated, all rat groups showed a gradual rise in choosing the large-reward arm from day 1 to day 3 of the learning phase. In the testing phase, SHR control rats opted for the large but delayed reward arm significantly less often (*p <* 0.001) than WKY control rats. Administration of MDL or IPS led to a significant increase (*p <* 0.001) in the preference for the large, delayed-reward arm in SHR rats, exceeding the selection rates seen in both SHR and WKY control groups (Fig. **4A**). During the testing phase, WKY control rats exhibited a significantly higher (*p <* 0.001) average percentage of selections for the large, delayed-reward arm compared to their SHR counterparts. Furthermore, juvenile administration of MDL or IPS in SHR rats led to a notable increase (*p <* 0.001) in the preference for the delayed reward option relative to both SHR and WKY control groups (Fig. **4B**). These results indicate that early pharmacological intervention can effectively reverse impulsive and disinhibited behavior patterns observed in young adult SHR rats.

### 3.5 Impulsive Response Measurement in the Electro Foot Shock Water Task

**Fig. (5):**
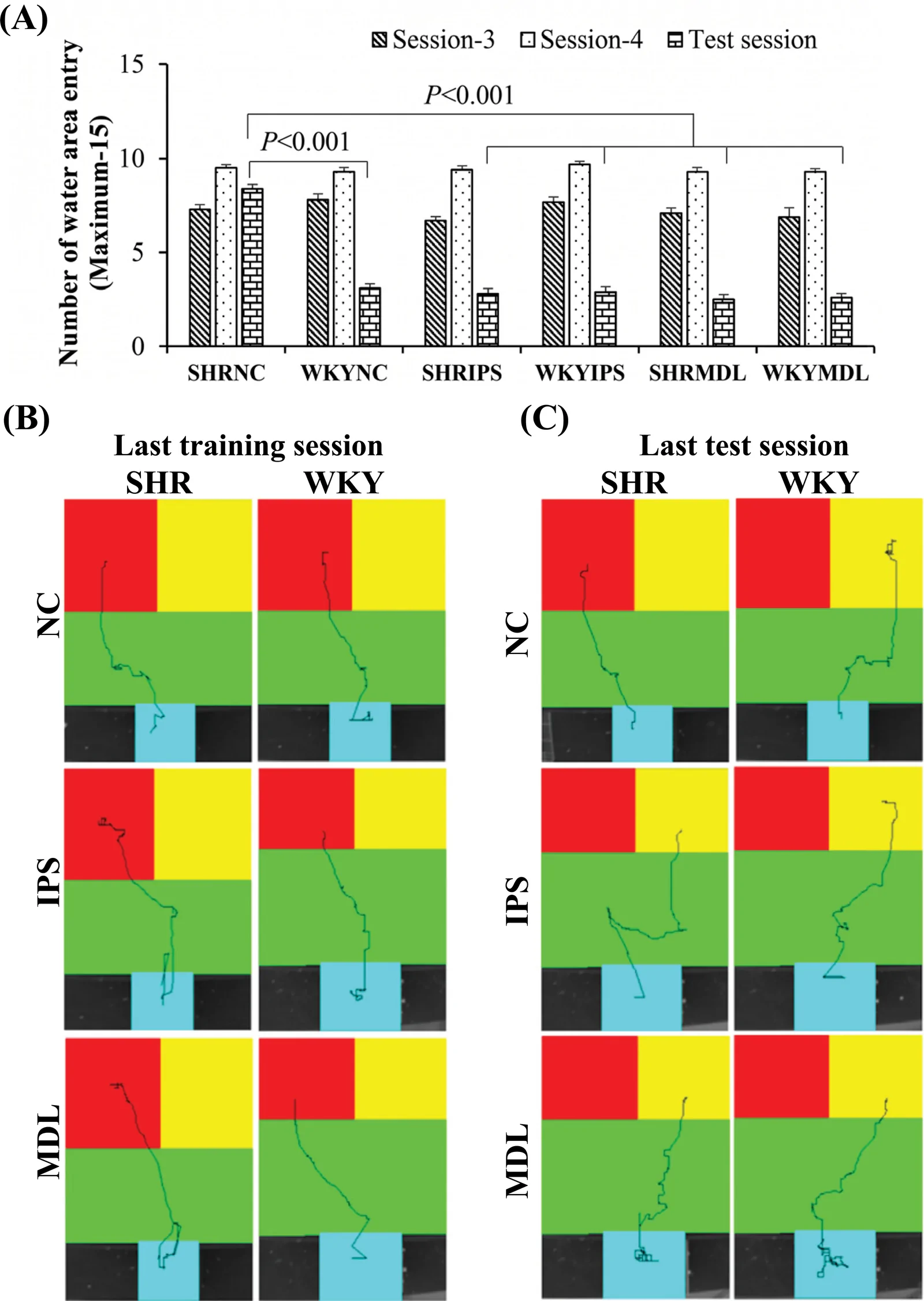
(**A**). Frequency of entries into the water zone during the 3^rd^ and 4^th^ training sessions and the impulsivity test session in the aversive electro-foot shock setup. SHR-NC rats showed significantly more water area entries, despite receiving a shock on each drinking attempt, indicating greater impulsive drinking than WKY-NC. In contrast, SHR rats treated with IPS or MDL spent significantly less time in the water area than SHR-NC. (**B**). Sample video tracking during the last training session- over 90% of rats from all groups entered the water area. (**C**). Sample video tracking during the last test session-SHR rats treated with IPS or MDL did not enter the water area as SHR-NC rats did. (Color codes: Blue = Start area, Green = Choice area, Red = Water area, Yellow = No water area, n=12).

In the test phase, SHR control rats demonstrated a significantly greater number of entries into the water zone to drink, despite receiving foot shocks, compared to WKY controls (*p <* 0.001). Administration of MDL or IPS led to a marked reduction (*p <* 0.001) in water area entries in both SHR and WKY groups (Fig. **5A**). Sample video tracking images are presented in Fig. (**5B** and **5C**). These findings indicate that neonatal treatment with MDL or IPS effectively reduces the impulsive behavior observed in young adult SHR rats.

### 3.6 Regional Neuroreceptor Expression and Immunohistochemical Localization

#### 3.6.1 Prefrontal Cortex

**Fig. (6):**
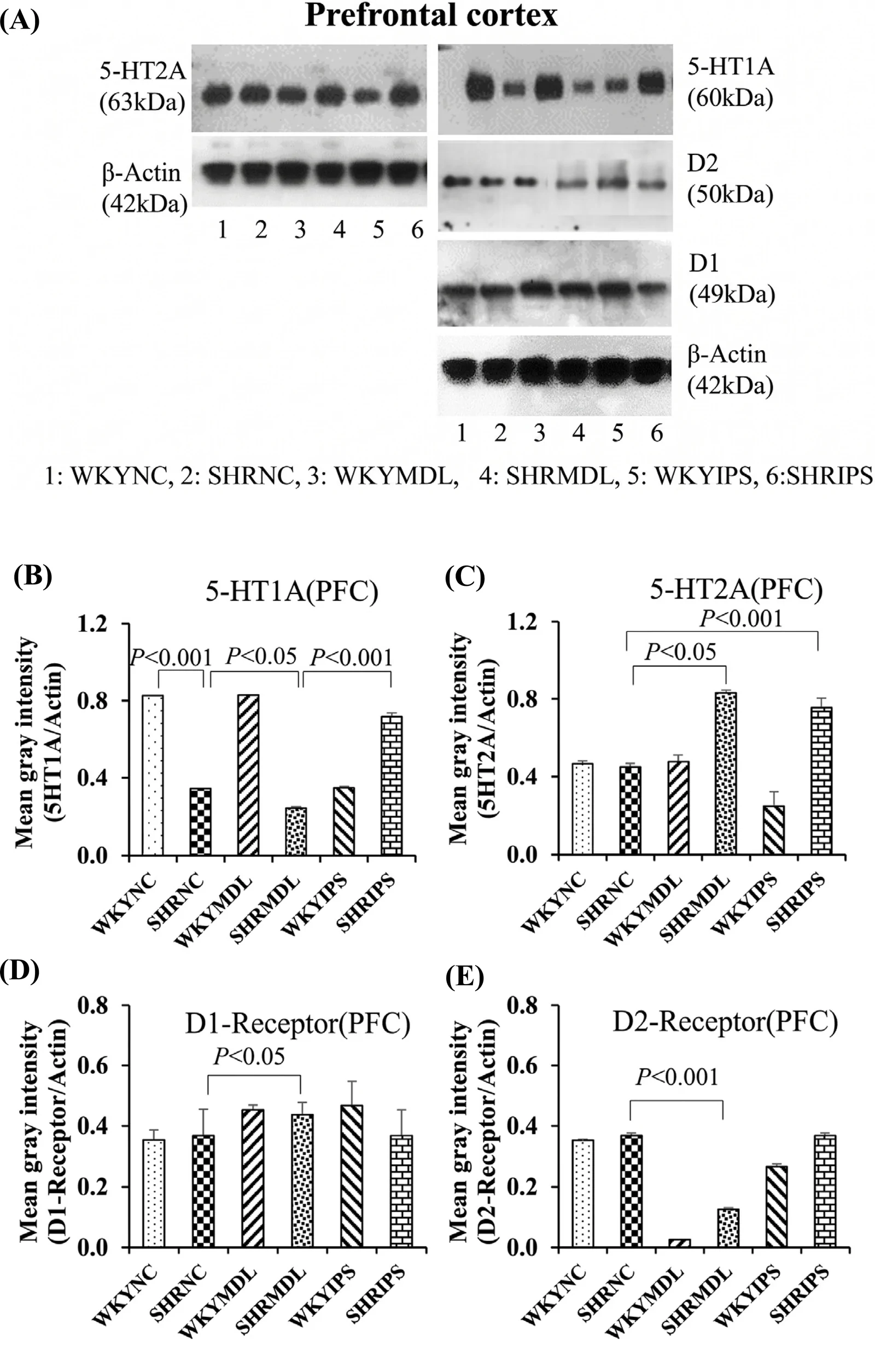
(**A**) Immunoblots of 5-HT1A, 5-HT2A, Dopamine-D1 and dopamine-D2- D2 receptors in the prefrontal cortical tissues. (**B-E**) Mean gray intensity of 5-HT1A, 5-HT2A, D1, and D2 receptors immunobands normalized to actin band gray intensity (n=6).

Initially, SHR control rats exhibited markedly reduced 5-HT1A receptor expression compared to WKY controls (*p <* 0.001). In contrast, 5-HT2A receptor levels did not differ significantly between the two groups (*p >* 0.05). Administration of IPS (a 5-HT1A receptor agonist) or MDL (a 5-HT2A receptor antagonist) in SHR rats led to a significant increase (*p <* 0.001) in the expression of 5-HT2A receptors. Conversely, IPS treatment in WKY rats caused a decrease in 5-HT1A receptor levels relative to untreated WKY controls. At baseline, there were no significant differences in DA-D1 or DA-D2 receptor expression between SHR and WKY control rats. Administration of IPS had no effect on the expression levels of either receptor in SHR rats compared to their untreated counterparts. Notably, treatment with MDL, a 5-HT2A receptor antagonist, led to a significant increase in DA-D1 receptor levels (*p <* 0.05) and a marked decrease in DA-D2 receptor expression (*p <* 0.001) in SHR rats (Fig. **6**). The immunohistochemical labeling pattern observed for 5-HT2A, 5-HT1A, DA-D1, and DA-D2 receptors across groups (Fig. **6**) aligns with the average gray intensity values obtained from the Western blot analysis presented in Fig. (**7** to **10**).

#### 3.6.2 Striatum

Initially, SHR control rats exhibited a significantly lower expression of the 5-HT1A receptor compared to WKY controls (*p <* 0.001). Administration of either IPS (a 5-HT1A receptor agonist) or MDL (a 5-HT2A receptor antagonist) led to a notable increase in 5-HT1A receptor levels (*p <* 0.001). In contrast, baseline expression of the 5-HT2A receptor did not differ significantly between SHR and WKY control rats. Although MDL treatment had no impact on 5-HT2A receptor expression in SHR rats, those treated with IPS showed a slight but significant increase (*p <* 0.05) in receptor levels compared to untreated SHR rats. Furthermore, SHR rats that received either MDL or IPS treatment showed a significant reduction in DA-D1 receptor expression (*p <* 0.05) when compared to untreated SHR controls. At the initial baseline level, no notable difference in DA-D1 receptor expression was observed between SHR and WKY control groups. However, DA-D2 receptor levels were significantly decreased (*p * 0.001) in SHR control rats relative to their WKY counterparts. In SHR rats, administration of either MDL or IPS led to a marked increase in DA-D2 receptor expression (*p * 0.001) compared to untreated SHR controls (Fig. **6**). The receptor distribution patterns visualized through immunostaining (Fig. **6**) for 5-HT2A, 5-HT1A, DA-D1, and DA-D2 closely matched the average band intensity results obtained from Western blot analysis, as illustrated in Figs. (**11** to **15**).

### 3.7 Regional Dopamine Concentration in the PFC and Striatum

**Fig. (16):**
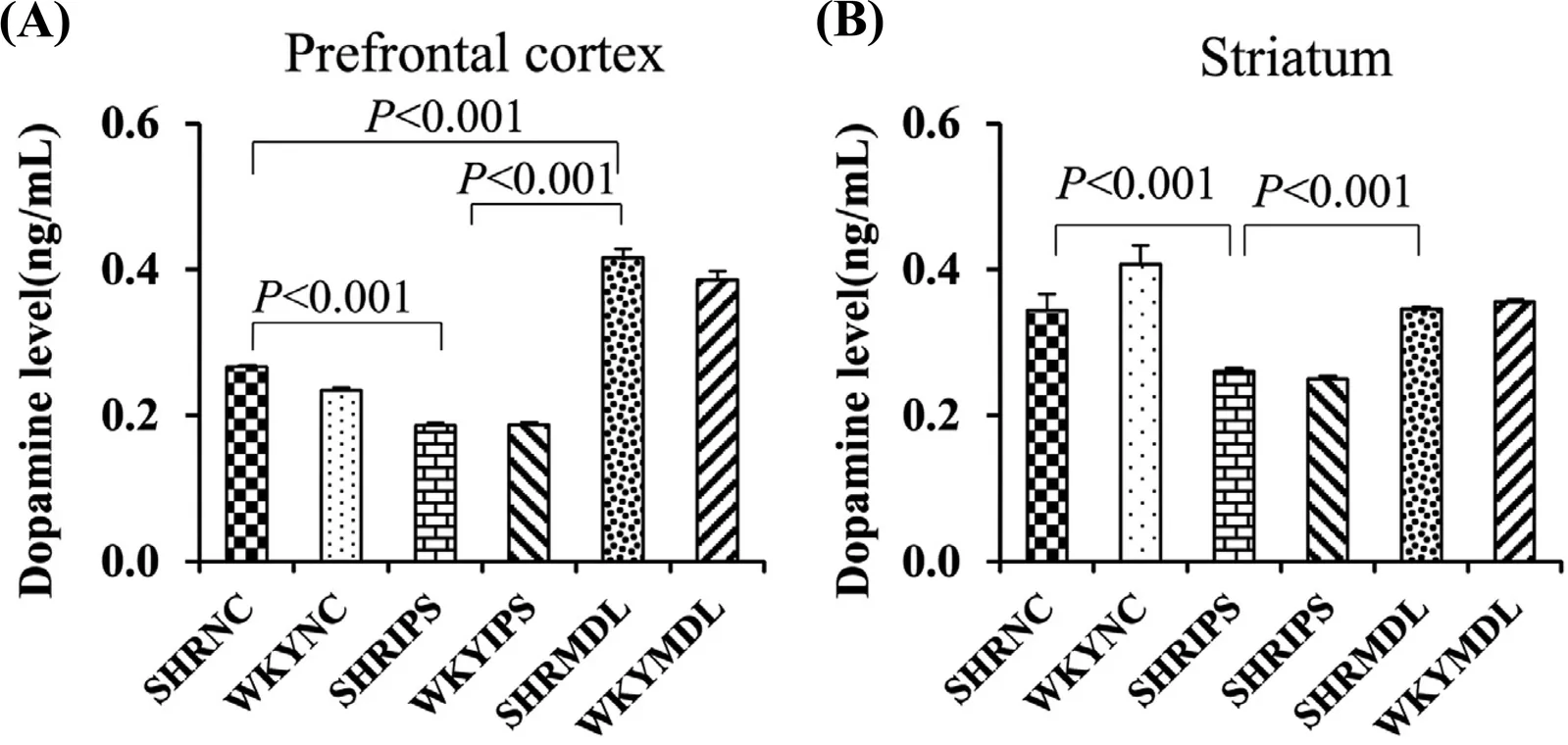
Mean dopamine concentrations in the prefrontal cortex (**A**) and striatum (**B**) of rat brain homogenates (n=6), measured using a standard ELISA kit and expressed in ng/ml homogenate. IPS treatment in SHR rats reduced dopamine levels in both regions, while MDL treatment increased dopamine levels in the prefrontal cortex but not in the striatum. No differences in dopamine levels were observed between SHRNC and WKYNC rats in both regions (n=6).

Baseline dopamine concentrations in both the prefrontal cortex and striatum showed no significant differences (*p * 0.05) between SHR and WKY rats. However, administration of the 5-HT1A agonist IPS in SHR rats led to a significant decrease in dopamine levels (*p * 0.001) in both brain regions compared to untreated SHR and WKY control groups. Administration of the 5-HT2A antagonist MDL in SHR rats significantly elevated dopamine levels in the prefrontal cortex (*p * 0.001), while dopamine levels in the striatum remained unchanged (*p * 0.05) relative to SHR controls (Fig. **16**). Additionally, MDL-treated SHR rats exhibited notably higher dopamine concentrations in both the prefrontal cortex and striatum when compared to those treated with IPS (*p * 0.001).

## 4 DISCUSSION

 The primary behavioral symptoms of ADHD, excessive activity, lack of attention, and impulsive actions, are exhibited by SHR. They show hyperlocomotor activity, with increased movement counts and velocity compared to WKY controls. Both 5-HT2A agonist and 5-HT1A antagonist treatments reduced these behaviors, with lasting effects into adulthood. Dysregulated serotonin pathways may contribute to hyperactivity in ADHD [43]. Serotonin, crucial for mood and behavior, affects impulsivity and hyperactivity through 5-HT2A and 5-HT1A receptors. Striatal 5-HT1A receptors, which regulate DA release and motor behavior [44], are essential for motor control, and their dysfunction can lead to hyperactivity. The 5-HT2A receptor also impacts DA release and is linked to impulsive and hyperactive behaviors [45]. In this study, SHR showed lower 5-HT1A receptor expression in the striatum and PFC compared with WKY controls, suggesting 5-HT1A downregulation in ADHD. Although 5-HT2A receptor dysfunction can affect DA regulation, no notable variation in its expression was found between the SHR and WKY control groups. However, 5-HT1A agonist and 5-HT2A antagonist treatments effectively reduced hyperlocomotor activity, evidenced by decreased movement counts and velocity, especially during prolonged open-field testing. Dysregulated DA transmission, particularly in the striatum and PFC, is strongly related to hyperactivity and impulsivity in ADHD [46]. DA-D2 receptors in the PFC control motor and reward processes, while DA-D1 receptors in the striatum influence motor control and attention [47, 48]. Dysfunction of these receptors can impair the inhibition of hyperactivity. Additionally, striatal DA-D2 receptor dysfunction may lead to excessive motor activity and reward-driven hyperactivity. In this study, striatal expression of the DA-D2 receptor was markedly reduced in SHRs compared with WKY controls; however, DA-D1 receptor expression in both the striatum and the prefrontal cortex showed no significant difference. This suggests that striatal DA-D2 dysregulation may contribute to hyperlocomotor activity.

Although the 5-CSRTT is the most reliable test for assessing attentional ability, we demonstrated that disinhibited behaviors in young SHRs could be reversed by either a 5-HT1A antagonist or a 5-HT2A agonist in the impulsivity (tolerance delay) test using a modified T-maze. This beneficial effect persisted into adulthood, suggesting long-term benefits. 5-HT receptors have a crucial function in mood regulation, cognitive processing, and attention. Dysregulation of serotonin signaling, particularly through 5-HT2A and 5-HT1A receptors, can impair attention [49]. Morici *et al*. [50] implicated 5-HT2A receptors in ADHD and other neuropsychiatric disorders, including schizophrenia and Alzheimer's. Disruptions in this system can affect sustained attention and cognitive flexibility [27]. As mentioned, 5-HT1A expression was notably reduced in the striatum and PFC of SHRs compared to WKY controls, indicating that reduced 5-HT1A activity contributes to hyperactivity and impulsivity in SHRs. The observed benefits were primarily linked to enhanced 5-HT2A receptor activity in the striatum and prefrontal cortex. While 5-HT1A stimulation upregulated its receptor and alleviated ADHD symptoms, 5-HT2A blockade reduced 5-HT1A receptor expression in the prefrontal cortex but upregulated it in the striatum. The data suggest that upregulating striatal and PFC 5-HT2A receptor activity may reduce ADHD-like behaviors in SHRs. Overall, the beneficial behavioral effects were associated with increased 5-HT1A expression, except for the 5-HT2A blockade response in the prefrontal cortex. The prefrontal DA-D1 receptors are important for maintaining working memory, supporting cognitive flexibility, and attention. Dysfunction in DA-D1 receptor activity impairs focus and sustained attention [51]. In the PFC and striatum, DA-D2 receptors are associated with reward processing and cognitive function [52], and their dysregulation contributes to distractibility and attention deficits. In this study, both treatments reduced DA-D1 receptors and upregulated striatal DA-D2 receptors. While 5-HT1A agonist treatment did not affect the PFC, 5-HT2A blockade increased DA-D2 and reduced DA-D1 receptors. A recent study indicates that inhibition of DA-D2 or DA-D1 receptors in the prefrontal cortex leads to attention deficits in mice; DA-D2 receptor blockade also leads to increased neuronal activation in the striatum, as indicated by elevated c-Fos expression [53], suggesting the striatum's involvement in attention deficits. In our study, the reversal of attentional impairment correlates with enhanced striatal DA-D2 receptor expression.

The tendency toward impulsive actions, as noted in the impulsive water-drinking test in SHR, was improved by both serotonergic manipulations and persisted into adulthood. Stimulants known to augment dopamine signaling in the brain suggest DA’s role in impulsivity symptoms in ADHD [54]. Crosstalk between serotonergic and dopaminergic systems contributes to impulsivity [55] and may influence ADHD etiology [25]. DA-D1 receptors in the PFC and striatum regulate cognitive functions, attention, and impulse control [47]. DA-D1 dysfunction, particularly in the PFC, impairs impulse control and regulation of inappropriate actions [56]. Our study found no significant baseline differences in DA-D1 receptor expression in either region. Striatal DA-D2 receptors also play a role in reward processing and motor control, with dysregulation contributing to impulsivity by disrupting reward-seeking and inhibition [57]. In our study, SHR rats exhibited significantly reduced DA-D2 receptor expression in the striatum compared with WKY control rats, but no difference in the PFC. Both 5-HT1A and 5-HT2A receptors modulate mood and inhibit impulsivity [45], and impairment in their regulation has been associated with impulsive behavior and poor emotional control. The opposing roles of 5-HT2A and 5-HT1A receptors in executive control and attentional functioning [58] suggest their involvement in impulsivity and compulsive behavior. Among the many serotonergic receptors, the 5-HT1A receptor is linked with impulsivity and anxiety, with genetic variations linked to ADHD risk [59]. In this study, SHR rats showed reduced 5-HT1A receptor expression relative to WKY control rats, but no baseline difference was observed for 5-HT2A receptors. Our findings suggest that 5-HT1A receptors contribute to ADHD-like behaviors in SHR. The reversal of impulsivity with 5-HT1A agonist treatment was associated with increased expression of both 5-HT1A and 5-HT2A receptors in the striatum and PFC. Interestingly, 5-HT2A antagonist treatment resulted in reduced 5-HT2A receptor expression in the prefrontal cortex, while being negligible in the striatum. Furthermore, 5-HT2A antagonist treatment downregulated 5-HT1A expression in the PFC while upregulating its expression in the striatum. A study confirmed that administration of a 5-HT1A agonist enhances impulsivity in both delay discounting tasks and reaction time, and this response is reversible with a 5-HT2A receptor blocker [60]. The opposing effects of serotonin on impulsivity are further supported by research showing that a 5-HT1A agonist reduces impulsivity in a choice reaction time task [61] but increases it in delay discounting. Moreover, it has been demonstrated that a selective 5-HT2A antagonist can decrease impulsivity in the 5-CSRTT [62, 63]. Moreover, blocking PFC 5-HT2A receptors reduces impulsive behavior [58]. While there is no evidence that the 5-HT1A agonist buspirone affects impulsivity in humans [64], the 5-HT2A antagonist quetiapine has demonstrated effectiveness in minimizing impulsivity [65]. Apart from DA and NE, a number of investigations have found lower levels of 5-HT in pediatric ADHD populations compared to healthy counterparts [43, 64]. Though we could not estimate 5-HT levels in this study, we demonstrate that enhancing 5-HT activity, either through a 5-HT1A agonist or a 5-HT2A antagonist, alleviates core ADHD symptoms in spontaneously hypertensive rats (SHR), likely by indirectly modulating DA activity.

### 4.1 5-HT1A Receptors

5-HT1A receptors are predominantly located on the cell bodies and dendrites of serotonergic neurons within the dorsal and median raphe nuclei, where they function as autoreceptors to control the production and release of serotonin [66, 67]. Postsynaptic expression of these receptors is also observed in regions such as the nucleus accumbens and PFC, where they interact with DA neurons of the mesocortical and mesolimbic pathways involved in ADHD. Additionally, 5-HT1A receptors are found in the midbrain Ventral Tegmental Area (VTA), the origin of DA neurons in both pathways [68]. Previously, it was observed that long-term administration of a 5-HT1A agonist during early life alleviated ADHD-like behaviors in young rats by increasing PFC 5-HT1A receptor expression and reducing it in the striatum [31]. In this study, a similar treatment had beneficial effects on ADHD behaviors, suggesting potential long-term benefits. Moreover, there was an increase in 5-HT1A receptor expression in the striatum during adulthood, a change not observed in young rats. The prefrontal cortex and striatal 5-HT2A receptor expressions were also upregulated, mirroring findings in young SHR rats.

### 4.2 5-HT1A Receptors and DA

Compared to our earlier research, DA-D1 receptor expression in the prefrontal cortex remained unchanged, although a decrease was observed in young SHR rats. The DA-D1 and DA-D2 receptor expression in the striatum was consistent with what we observed in young rats. Systemic delivery of a 5-HT1A agonist, which activates these receptors, is known to affect the DA neurotransmission *via* at least two mechanisms [69]. a) 5-HT availability in the soma and terminal regions of DA pathways is diminished following the activation of somatodendritic 5-HT1A receptors, which in turn reduces the 5-HT inhibitory effects on the release of DA. This autoreceptor-facilitated modulation is vital for nigrostriatal DA neurotransmission, as 5-HT1A receptors are more densely concentrated in the Dorsal Raphe (DR) nucleus than in the Median Raphe (MR), with the Substantia Nigra (SN) receiving serotonergic input primarily from the DR. b) Postsynaptic activation of 5-HT1A receptors in regions including PFC and VTA also promotes DA neurotransmission. The effects of postsynaptic receptors appear particularly important for mesocortical DA systems, given the abundant presence of 5-HT1A receptors in the PFC. In our study, prolonged treatment with a 5-HT1A receptor agonist led to a decrease in DA levels in the PFC. This decrease was associated with a reduction in PFC DA-D2 receptors and an increase in striatal DA-D2 receptors. A Gi-protein-coupled receptor, the 5-HT1A receptor, inhibits adenylyl cyclase upon activation, lowering cAMP levels. This reduces protein kinase A (PKA) activity, which in turn can decrease ion channel activation and DA release [70]. Additionally, presynaptic 5-HT1A receptors on dopaminergic terminals inhibit DA release by decreasing neurotransmitter release from the presynaptic neuron [70]. This acts as a brake on DA release, lowering DA levels. Furthermore, 5-HT1A receptors can indirectly affect DA release by interacting with other neurotransmitter systems, such as serotonin or GABA, which modulate dopaminergic neuron activity. As serotonin autoreceptors, 5-HT1A receptors inhibit further serotonin release, disrupting the balance between serotonin and DA, which may further lower DA levels in the PFC. To summarize, stimulation of 5-HT1A receptors leads to a direct reduction in DA release and, indirectly, to presynaptic mechanisms and reduced dopaminergic neuron activity in the PFC.

### 4.3 5-HT2A Receptors

While 5-HT2A antagonists, especially atypical antipsychotics, have been used in pediatric populations, comprehensive long-term studies on their chronic use in children are lacking. The major serotonergic neurons that ascend from the MR and DR nuclei establish extensive connections with structures involved in ADHD, including the VTA, substantia nigra, hippocampus, amygdala, and PFC [71, 72]. 5-HT2A receptors in the cortical region are primarily found in the pyramidal cell’s apical dendrites [73], with most being postsynaptic [74]. Serotonin modulates multiple neurotransmitter systems at the synapse, including DA neurons located in the VTA and substantia nigra [75, 76]. Additionally, the interplay between dopamine and serotonin is considered to play a role in impulsivity and may exert a role in ADHD development [77, 78]. In our previous study, chronic treatment with MDL (the 5-HT2A antagonist) in juvenile SHR rats alleviated ADHD behaviors, and in the current study, we observed long-lasting improvements in ADHD symptoms in adult rats. However, the expression of 5-HT1A receptors differed from our previous findings in young rats. Specifically, in this study, adult rats exhibited reduced expression of 5-HT1A receptors in the prefrontal cortex, whereas it remained unaffected in juvenile rats. The striatal 5-HT1A receptor expression was increased in adults, in contrast to the downregulation seen in juvenile rats in our previous report. The expression of 5-HT2A receptors in the PFC of adult rats was similar to that in young rats. Administration of a 5-HT2A antagonist led to increased 5-HT2A receptor expression in the striatum of juvenile rats, whereas adult rats showed no such alteration. In a recent study by Lin *et al*. [79], the amelioration of hyperactivity in SHR after dexamethasone (a Glucocorticoid receptor agonist) was associated with elevated 5-HT2A receptor expression and decreased 5-HT1A receptor expression in the PFC, which is parallel to our present findings.

### 4.4 5-HT2A Receptors and DA

Comparing our results with previous work in young rats, we found that DA-D1 and DA-D2 receptor countenance in the striatum and PFC remained similar between young and adult rats, except for an upregulation of PFC DA-D1 receptors in adults. In conclusion, MDL, the 5-HT2A antagonist treatment in juvenile rats, led to lasting improvements in ADHD behaviors in adulthood, along with significant changes in serotonin receptor expression. The 5-HT2A receptor interacts with DA systems [76, 80]. Serotonergic neurons directly synapse onto neurons in the VTA, including DA neurons, thereby modulating their output in various ways [81]. For example, chronic fluoxetine administration reduces DA in the striatum [82, 83] but increases it in the PFC [84]. Enhancement of DA release by activation of the 5-HT2A receptor is reported. Blocking 5-HT2A receptors can indirectly enhance DA release in the PFC [76]. The 5-HT2A receptor enhances neuronal activity in VTA and DA release [85]. Prefrontal pyramidal and non-pyramidal neurons both express 5-HT2A and DA-D2 receptors, and they share common signaling pathways [86, 87].

In conclusion, these results indicate that the coordination between DA-D2 and 5-HT2A receptors influences neuronal activity *via* multiple pathways, highlighting the importance of maintaining balanced receptor activation. Additionally, atypical antipsychotics strongly bind to 5-HT2A receptors [88] and especially bind to 5-HT2A over DA-D2 receptors [89]. The increased binding affinity for 5-HT2A receptors is thought to potentiate the action of DA-D2 antagonists, thereby enabling therapeutic benefits at lower doses. The 5-HT2A receptor may influence dopamine release indirectly by regulating excitatory inputs from the PFC. Activating 5-HT2A receptors raises dopamine levels in the PFC, an effect prevented by selective antagonists [85, 90]. In the medial PFC, 5-HT2A receptors modulate VTA dopamine neurons, and their activation increases DA activity and release in the mesocortical pathway implicated in ADHD.

In our study, MDL treatment, a 5-HT2A antagonist, increased PFC DA levels without affecting striatal DA levels. This increase in PFC DA was accompanied by upregulation of D1 receptors and downregulation of D2 receptors. The differential effects on DA levels can be attributed to the differing functions of 5-HT2A receptors across these brain areas. 5-HT2A receptors are abundantly expressed in the PFC and inhibit DA release upon activation [91]. By blocking these receptors with MDL, the inhibition is removed, leading to increased DA release. In contrast, striatal 5-HT2A receptors are less abundant and may have a limited influence on DA release regulation. Thus, striatal blocking of 5-HT2A receptors has a minimal effect on DA levels. The PFC is more sensitive to serotonergic modulation, so blocking 5-HT2A receptors enhances DA release, especially when other serotonin receptors, like 5-HT1A, are not inhibiting it. The striatum, however, likely has a more balanced regulation of DA, so the same blockade has less impact. While 5-HT2A receptor blockade in the striatum can reduce serotonin-mediated DA suppression [27], the overall effect is region-specific. Furthermore, MDL shows higher selectivity for 5-HT2A receptors, and activating these receptors in the cortex enhances DA release *via* mesocortical pathways, potentially by increasing glutamate release from cortical projections targeting the VTA [90].

The serotonergic system is crucial for brain development throughout childhood and adolescence, influencing processes like synaptic pruning, neurogenesis, and neural circuit maturation [92]. Long-term modulation of serotonin receptors, particularly in early brain development, could disrupt these processes, potentially leading to developmental delays or cognitive impairments. 5-HT2A receptors contribute to synaptic plasticity, a fundamental process for learning and memory. Chronic antagonism may impair synaptic changes, particularly during critical periods of brain plasticity. Prolonged inhibition of 5-HT2A receptors may disturb the equilibrium between serotonin and DA, potentially affecting mood, behavior, and emotional regulation in children [85]. This may lead to behavioral or psychiatric symptoms and result in anxiety-like behavior. However, our study did not observe anxiety-like behavior. Prolonged or repeated exposure to drugs affecting neuroreceptors can lead to functional or structural changes in those receptors. The main outcome of this research is that the beneficial effects against ADHD behavior persisted even after drug treatment ended, likely due to neuroreceptor adaptation. This continued benefit can be explained by mechanisms related to neuroplasticity and long-term brain changes. Antipsychotic drugs, particularly with extended use, may help “reset” or "reorganize" disrupted neural circuits in conditions like ADHD [93]. These structural and functional changes in the brain can persist even after treatment cessation. Over time, the brain may adjust by altering the sensitivity or number of DA receptors. In our study, D2 receptor levels continued to be reduced in the prefrontal cortex and elevated in the striatum. In summary, the continued benefit after stopping treatment is likely due to neural, molecular, and psychosocial adaptations that occurred during treatment. However, this effect is not universal, and symptom recurrence may require ongoing monitoring and, if necessary, reinitiating treatment.

Over the past few decades, there has been a notable rise in the prescription of psychiatric drugs for children, especially dopamine agonists (stimulants) used to treat ADHD and dopamine antagonists (antipsychotics) prescribed for mood, anxiety, and psychotic conditions. Research shows that stimulants primarily affect DA-D1 receptors but also impact DA-D2 receptors [94]. Antipsychotics mainly act through DA-D2 antagonism [95], though they are non-selective, binding to all five DA receptor types [95]. For example, hyperactivity in mesolimbic dopaminergic pathways overstimulates DA-D2 receptors, while hypoactivity in mesocortical pathways reduces stimulation of cortical DA-D1 receptors, contributing to negative symptoms [20]. While DA agonists and antagonists manage ADHD symptoms, they are linked to adverse effects such as psychiatric disturbances, motor issues, metabolic changes, and cardiovascular risks [16, 96, 97]. Aripiprazole, with its 5-HTA agonist, 5-HT2A antagonist, and DA-D2 partial agonist properties, has been shown to improve ADHD behaviors like attention, motivation, and reduce negative thinking [98]. A separate study found that combining a 5-HTA agonist, a 5-HT2A antagonist, and a DA-D2 partial agonist (aripiprazole) with methylphenidate markedly reduced irritability, depressive symptoms, anxiety, attention difficulties, social challenges, and reaction-time inconsistencies [99]. Our study suggests that 5-HT2A or 5-HT1A agonists may modulate DA pathway activity *via* cortical mechanisms, offering benefits without the adverse effects of long-term DA receptor blockade. The present study addresses several important gaps in ADHD research. Earlier work has focused on short-term serotonergic interventions, leaving the long-term effects on behavior and dopamine-serotonin (DA-5-HT) interactions poorly defined. Baseline abnormalities in key receptors, such as 5-HT1A and striatal D2, have also been inconsistently reported, and links between persistent behavioral recovery and specific receptor changes remain unclear. Our findings show that juvenile treatment with a 5-HT1A agonist or a 5-HT2A antagonist produces lasting reductions in hyperactivity and impulsivity in SHR, accompanied by region-specific receptor adaptations, such as increased striatal D2 receptor expression and altered 5-HT1A/5-HT2A receptor expression, which help explain the durable behavioral benefit. This work highlights the novel concept that indirect modulation of dopamine circuits through serotonergic pathways can provide long-term therapeutic effects without continuous drug exposure. Future studies should confirm these mechanisms by directly measuring extracellular neurotransmitters, using broader attentional assays, and extending the approach to different developmental stages, sexes, and ultimately human subjects.

## 5 STUDY LIMITATION

One limitation of the study was that the 5-Choice Serial Reaction Time Task, which is considered the gold-standard paradigm for assessing attentional performance, was not employed. In addition, direct estimation of extracellular serotonin (5-HT) and dopamine (DA) levels in the striatum could have provided deeper insight into the neurochemical basis of the receptor alterations observed in this study.

## CONCLUSION

1. Treatment with 5-HT1A agonists and 5-HT2A antagonists in juvenile SHR reduced ADHD behaviors, with effects lasting into adulthood, indicating long-term benefits.
2. The lasting effect may result from neuroreceptor adaptation, particularly D2 receptor upregulation in the striatum and downregulation in the PFC.
3. Reduced brain DA levels after 5-HT1A agonist treatment and increased prefrontal DA after 5-HT2A antagonist treatment correlate with altered D2 receptor activity in the striatum.
4. SHR's hyperactivity, inattention, and impulsivity are associated with reduced 5-HT1A receptor expression in the PFC and striatum.
5. The observed benefits are mainly due to the upregulation of 5-HT2A receptor expression.

## Figures and Tables

**Fig. (7):**
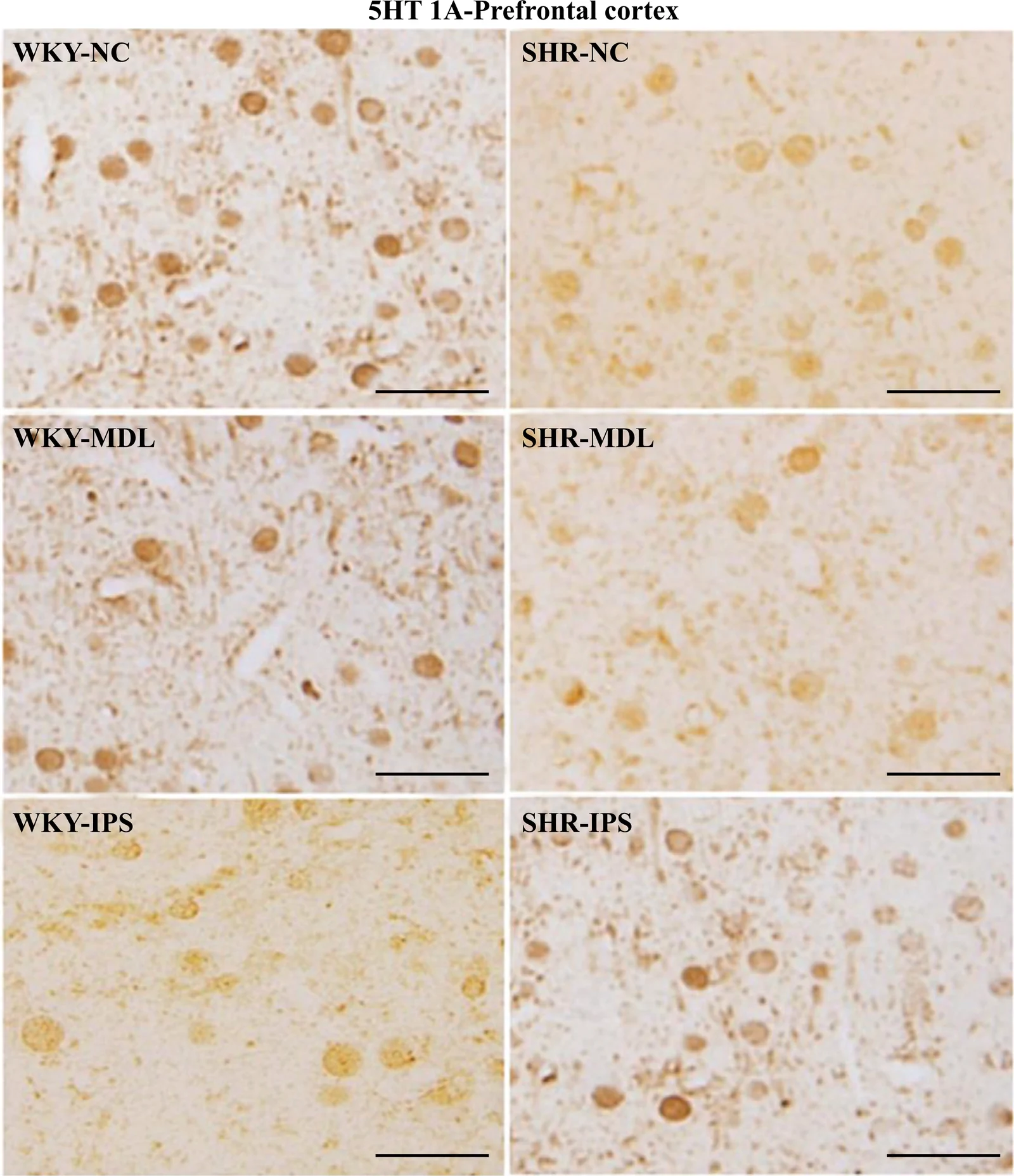
Representative images of prefrontal cortex sections showing immunostaining for 5-HT1A. The receptor expression patterns across different groups align with the mean gray intensities from the Western blot analysis shown in Fig. (**6B**). Scale bar: 50 µm.

**Fig. (8):**
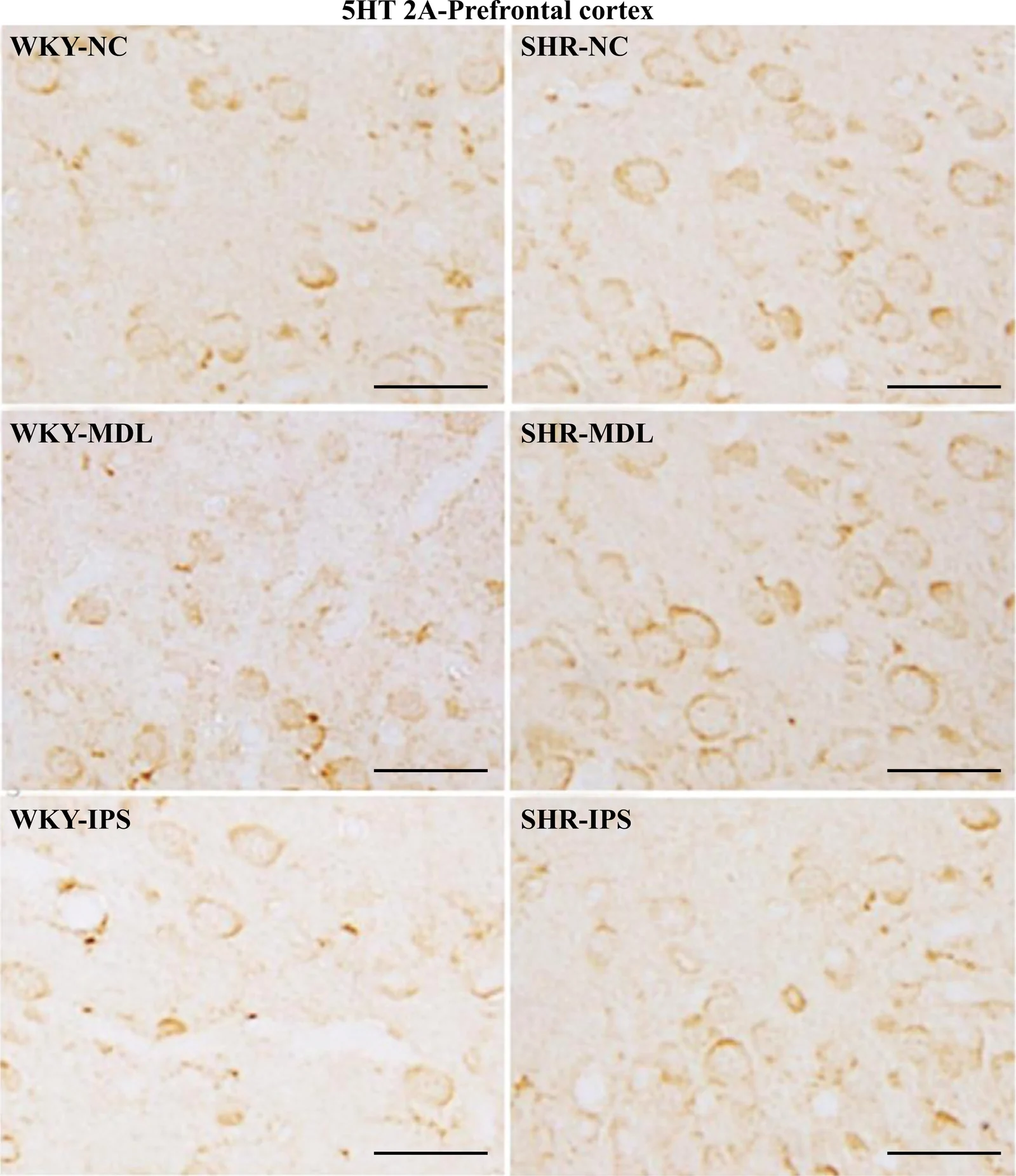
Representative images of prefrontal cortex sections showing immunostaining for 5-HT2A. The receptor expression patterns across different groups align with the mean gray intensities from the Western blot analysis shown in Fig. (**6C**). Scale bar: 50 µm.

**Fig. (9):**
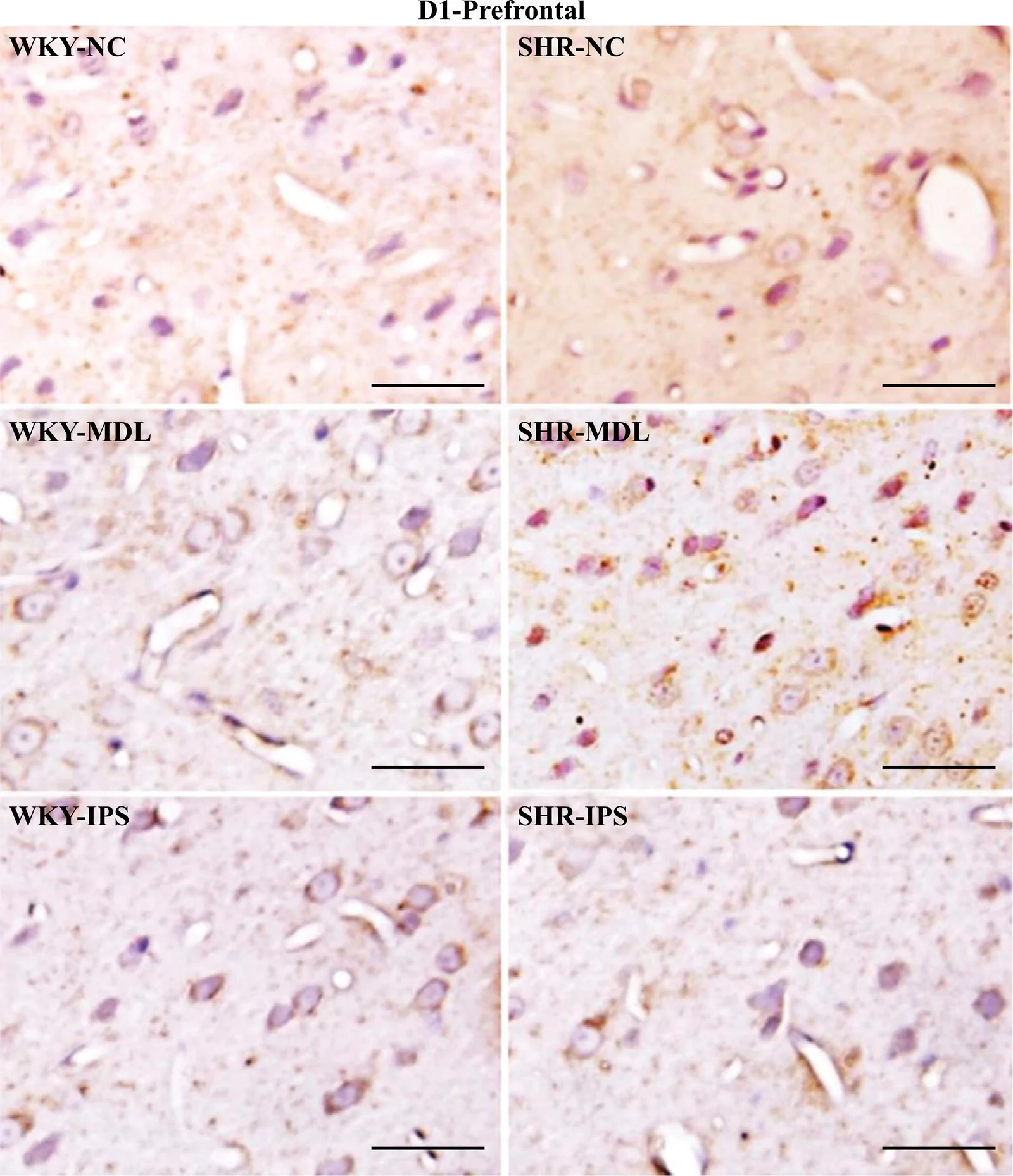
Representative images of prefrontal cortex sections showing immunostaining for D1 receptor (counterstained with hematoxylin stain). The receptor expression patterns across groups align with the mean gray intensities from the Western blot analysis shown in Fig. (**6D**). Scale bar: 50 µm.

**Fig. (10):**
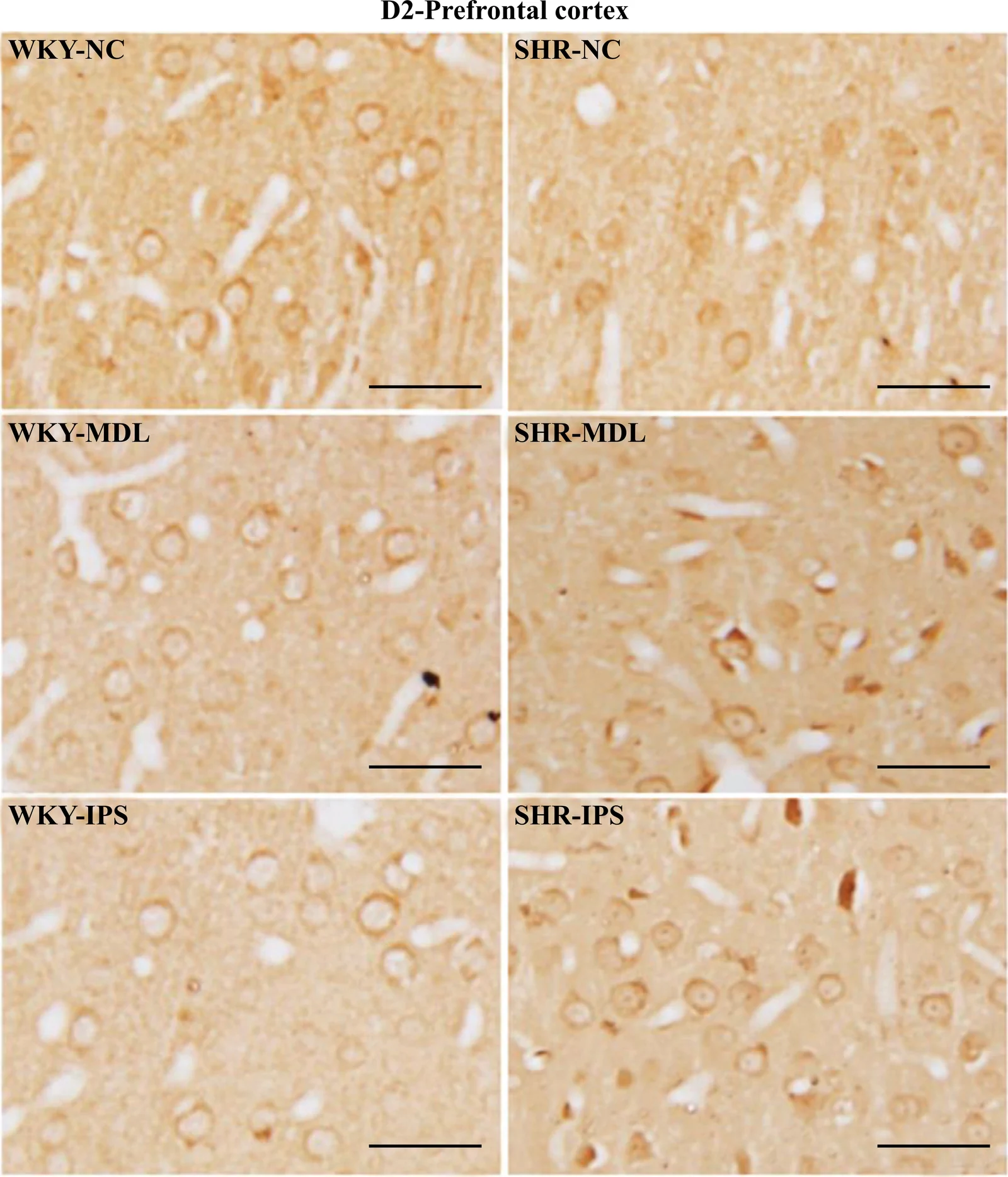
Representative images of prefrontal cortex sections showing immunostaining for D2 receptor. The receptor expression patterns across groups align with the mean gray intensities from the Western blot analysis shown in Fig. (**6E**). Scale bar: 50 µm.

**Fig. (11):**
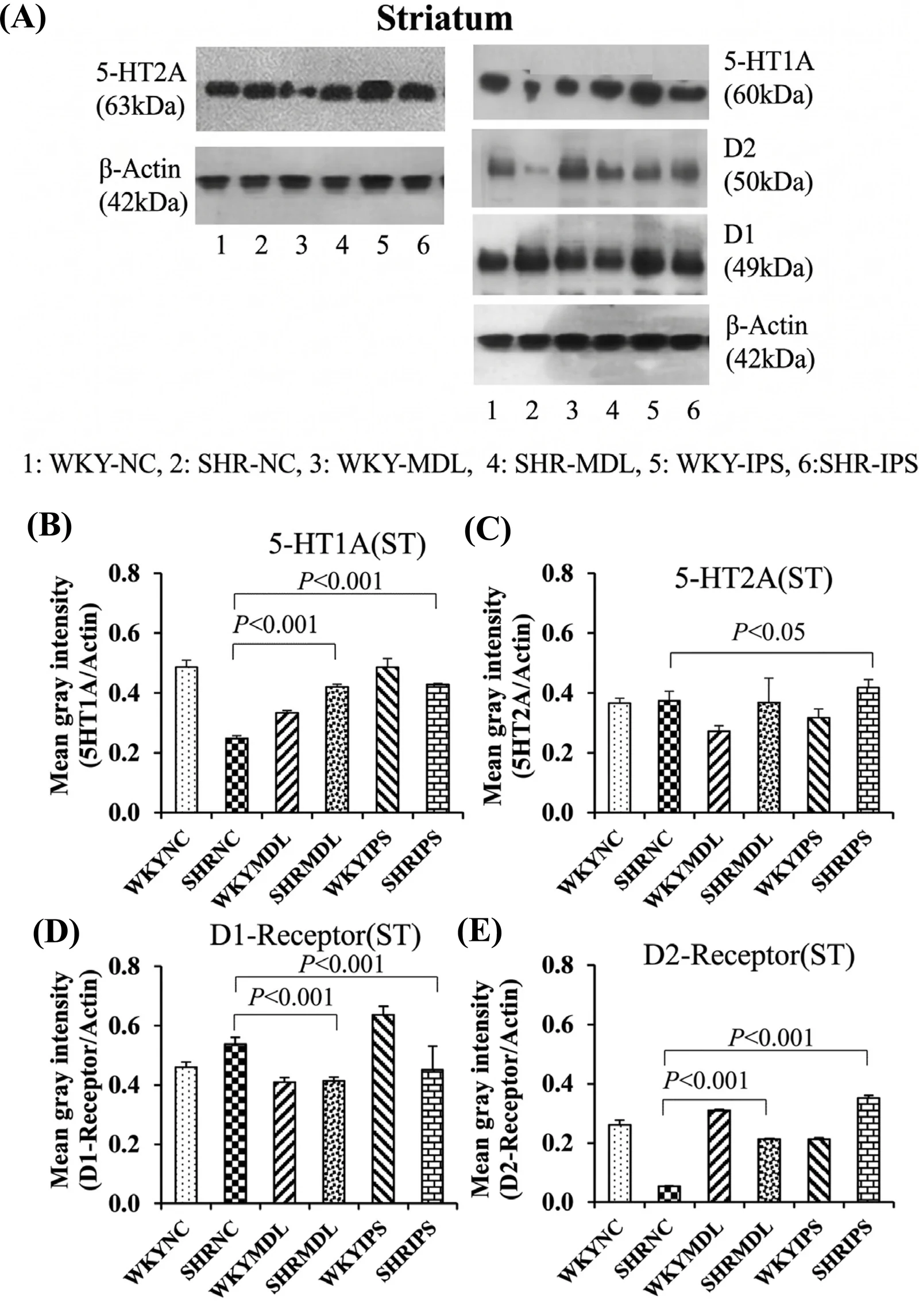
(**A**) Immunoblots of 5-HT2A, 5-HT1A, DA-D1, and DA-D2 receptors in the striatal tissues. (**B-E**) Mean gray intensity 5-HT2A, 5-HT1A, D1, and D2 receptors immunobands normalized to actin band gray intensity in the striatal tissues (n=6).

**Fig. (12):**
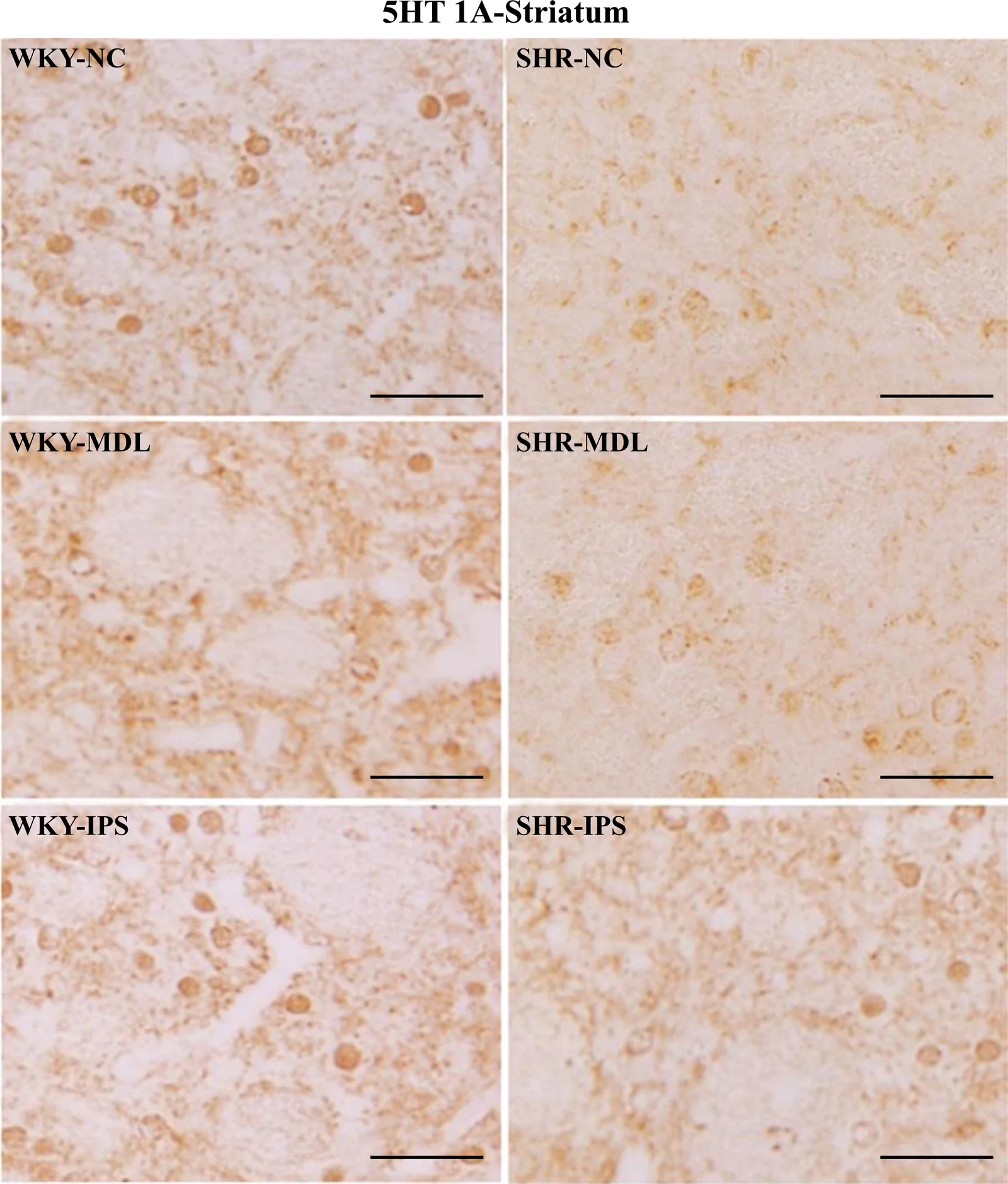
Representative photomicrographs of striatal brain sections showing immunostaining for 5-HT1A receptors. The observed receptor expression patterns across groups are consistent with the mean gray intensities obtained from the corresponding Western blot analysis shown in Fig. (**11B**). Scale bar: 50 µm.

**Fig. (13):**
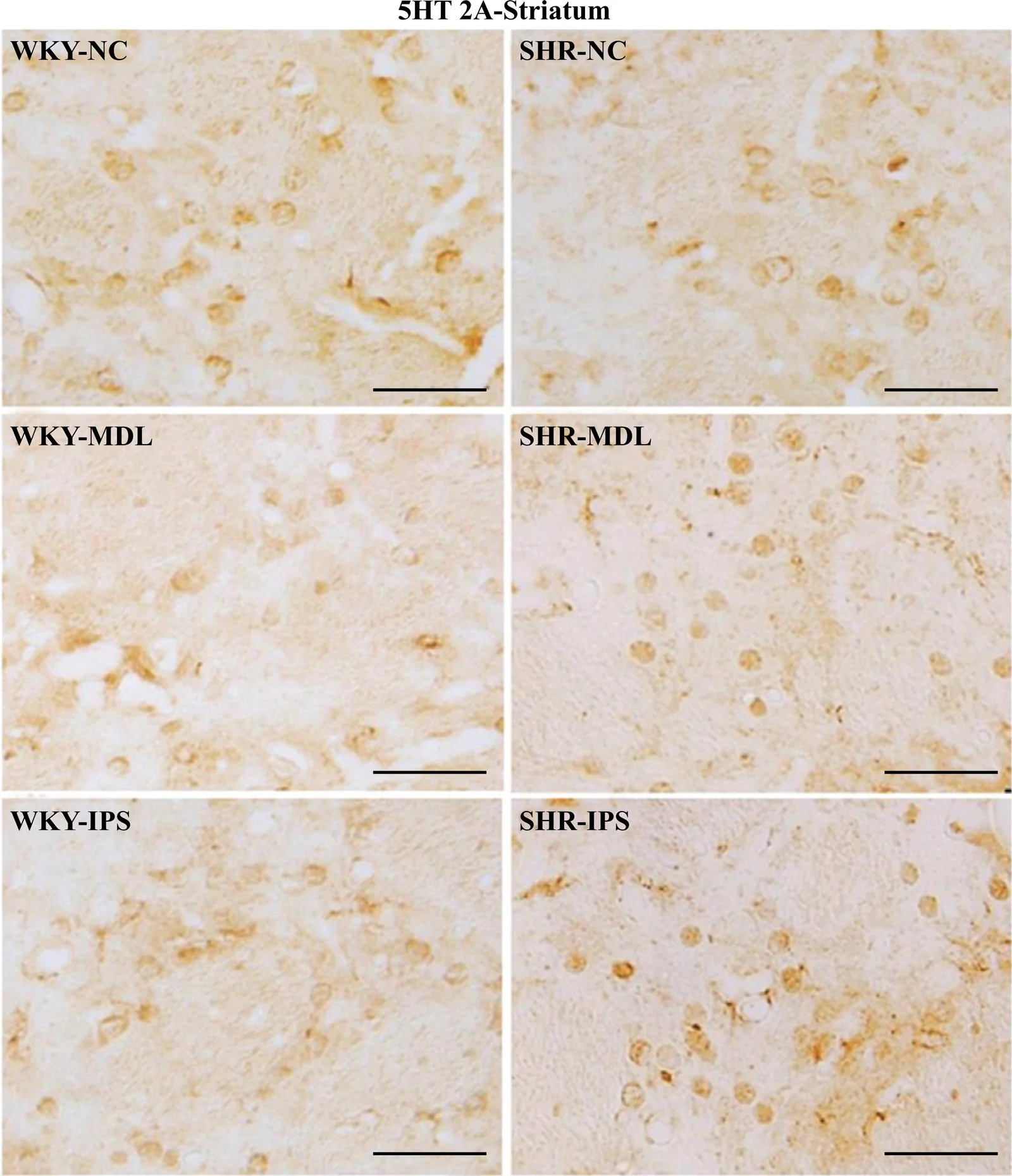
Representative photomicrographs of striatal brain sections showing immunostaining for 5-HT2A receptors. The observed receptor expression patterns across groups are consistent with the mean gray intensities obtained from the corresponding Western blot analysis shown in Fig. (**11C**). Scale bar: 50 µm.

**Fig. (14):**
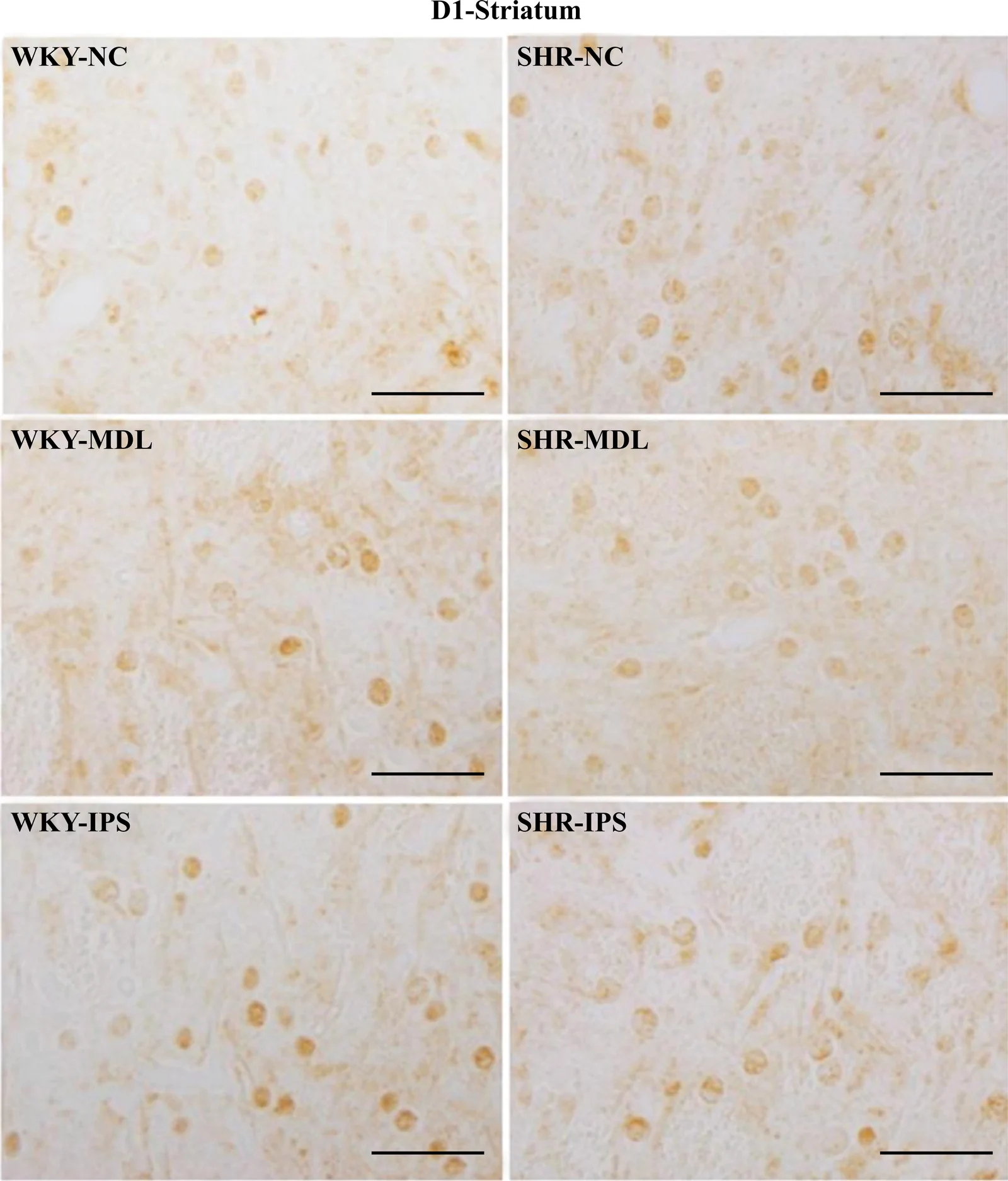
Representative photomicrographs of striatal brain sections showing immunostaining for D1 receptors. The observed receptor expression patterns across groups are consistent with the mean gray intensities obtained from the corresponding Western blot analysis shown in Fig. (**11D**). Scale bar: 50 µm.

**Fig. (15):**
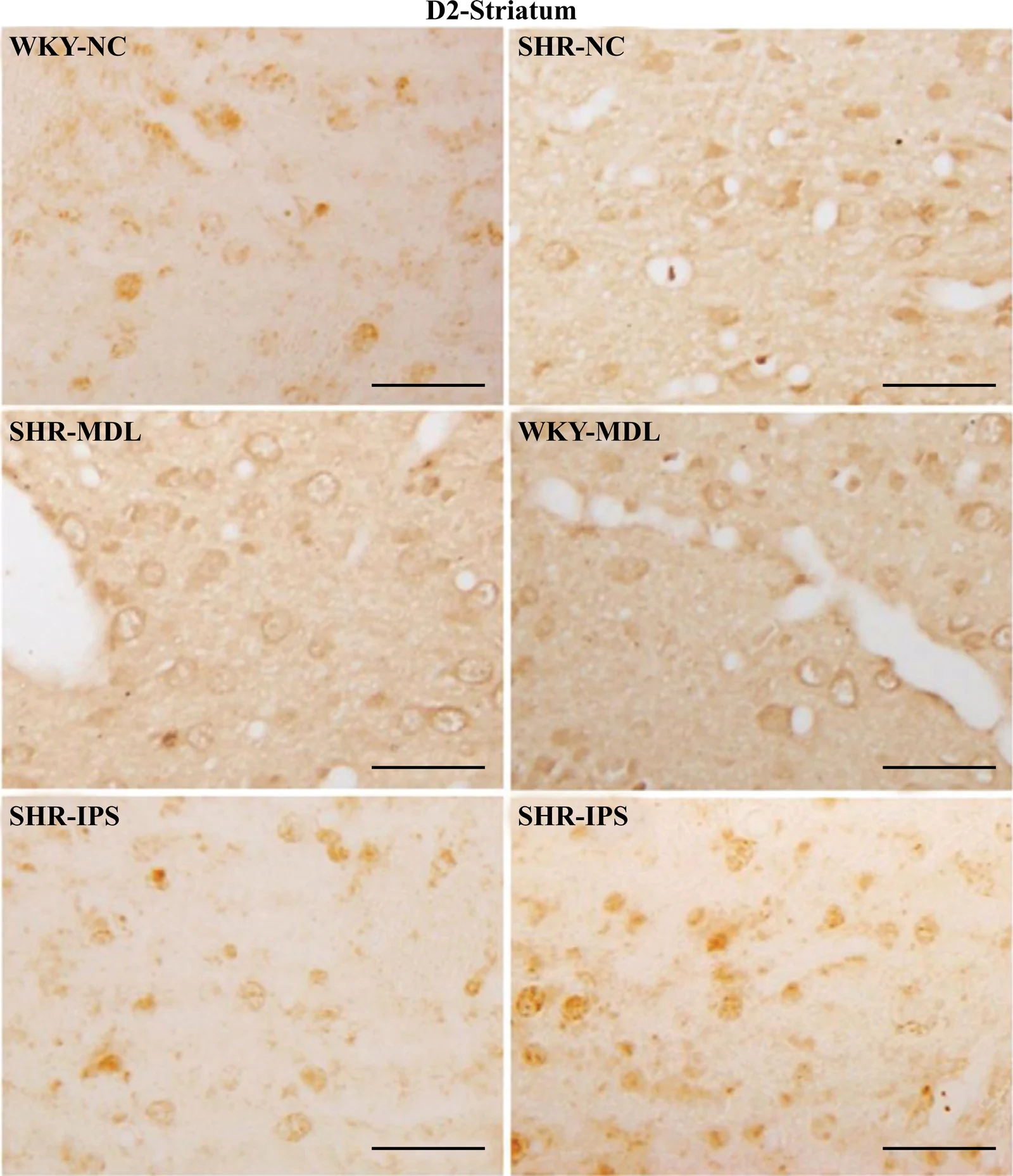
Representative photomicrographs of striatal brain sections showing immunostaining for D2 receptors. The observed receptor expression patterns across groups are consistent with the mean gray intensities obtained from the corresponding Western blot analysis shown in Fig. (**11E**). Scale bar: 50 µm.

## Data Availability

The data and supporting information are available in the article.

## LIST OF ABBREVIATIONS

ADHD: Attention Deficit Hyperactivity Disorder
DA: Reduced Dopamine
NE: Noradrenaline
PBS: Phosphate-Buffered Saline
PFC: Prefrontal Cortex
SHRs: Spontaneously Hypertensive Rats

## References

[1] Ayano G., Demelash S., Gizachew Y., Tsegay L., Alati R. (2023). The global prevalence of attention deficit hyperactivity disorder in children and adolescents: An umbrella review of meta-analyses.. J. Affect. Disord..

[2] Salari N., Ghasemi H., Abdoli N., Rahmani A., Shiri M.H., Hashemian A.H., Akbari H., Mohammadi M. (2023). The global prevalence of ADHD in children and adolescents: A systematic review and meta-analysis.. Ital. J. Pediatr..

[3] Danielson M.L., Claussen A.H., Bitsko R.H., Katz S.M., Newsome K., Blumberg S.J., Kogan M.D., Ghandour R. (2024). ADHD prevalence among U.S. children and adolescents in 2022: Diagnosis, severity, co-occurring disorders, and treatment.. J. Clin. Child Adolesc. Psychol..

[4] Giedd J.N., Rapoport J.L. (2010). Structural MRI of pediatric brain development: What have we learned and where are we going?. Neuron.

[5] Sobel L.J., Bansal R., Maia T.V., Sanchez J., Mazzone L., Durkin K., Liu J., Hao X., Ivanov I., Miller A., Greenhill L.L., Peterson B.S. (2010). Basal ganglia surface morphology and the effects of stimulant medications in youth with attention deficit hyperactivity disorder.. Am J. Psychiatry.

[6] Weyandt L., Swentosky A., Gudmundsdottir B.G. (2013). Neuroimaging and ADHD: FMRI, PET, DTI findings, and methodological limitations.. Dev. Neuropsychol.

[7] Firouzabadi F.D., Ramezanpour S., Firouzabadi M.D., Yousem I.J., Puts N.A.J., Yousem D.M. (2022). Neuroimaging in attention-deficit/hyperactivity disorder: Recent advances.. AJR Am J. Roentgenol.

[8] de Zeeuw P., Mandl R.C.W., Hulshoff Pol H.E., van Engeland H., Durston S. (2012). Decreased frontostriatal microstructural organization in attention deficit/hyperactivity disorder.. Hum. Brain Mapp.

[9] Pirot S., Godbout R., Mantz J., Tassin J.P., Glowinski J., Thierry A.M. (1992). Inhibitory effects of ventral tegmental area stimulation on the activity of prefrontal cortical neurons: Evidence for the involvement of both dopaminergic and GABAergic components.. Neuroscience.

[10] del Campo N., Chamberlain S.R., Sahakian B.J., Robbins T.W. (2011). The roles of dopamine and noradrenaline in the pathophysiology and treatment of attention-deficit/hyperactivity disorder.. Biol. Psychiatry.

[11] Biederman J., Spencer T. (1999). Attention-deficit/hyperactivity disorder (adhd) as a noradrenergic disorder.. Biol. Psychiatry.

[12] Arnsten A.F.T. (2000). Genetics of childhood disorders: XVIII. ADHD, Part. 2: Norepinephrine has a critical modulatory influence on prefrontal cortical function.. J. Am Acad. Child Adolesc. Psychiatry.

[13] Beaulieu J.M., Gainetdinov R.R. (2011). The physiology, signaling, and pharmacology of dopamine receptors.. Pharmacol. Rev..

[14] Fusar-Poli P., Rubia K., Rossi G., Sartori G., Balottin U. (2012). Striatal dopamine transporter alterations in ADHD: Pathophysiology or adaptation to psychostimulants? A meta-analysis.. Am J. Psychiatry.

[15] Faraone S.V. (2018). The pharmacology of amphetamine and methylphenidate: Relevance to the neurobiology of attention-deficit/hyperactivity disorder and other psychiatric comorbidities.. Neurosci. Biobehav. Rev..

[16] Nanda A., Janga L.S.N., Sambe H.G., Yasir M., Man R.K., Gogikar A., Mohammed L. (2023). Adverse effects of stimulant interventions for attention deficit hyperactivity disorder (ADHD): A comprehensive systematic review.. Cureus.

[17] Budur K., Mathews M., Adetunji B., Mathews M., Mahmud J. (2005). Non-stimulant treatment for attention deficit hyperactivity disorder.. Psychiatry.

[18] Pang L., Sareen R. (2021). Retrospective analysis of adverse events associated with non-stimulant ADHD medications reported to the United States Food and Drug Administration.. Psychiatry Res..

[19] Ahmed R., Aslani P. (2013). Attention-deficit/hyperactivity disorder: An update on medication adherence and persistence in children, adolescents and adults.. Expert Rev. Pharmacoecon. Outcomes Res..

[20] Abi-Dargham A., Laruelle M. (2005). Mechanisms of action of second generation antipsychotic drugs in Schizophrenia: Insights from brain imaging studies.. Eur. Psychiatry.

[21] Weintraub D., Koester J., Potenza M.N., Siderowf A.D., Stacy M., Voon V., Whetteckey J., Wunderlich G.R., Lang A.E. (2010). Impulse control disorders in Parkinson disease: A cross-sectional study of 3090 patients.. Arch. Neurol..

[22] Duma S.R., Fung V.S.C. (2019). Drug-induced movement disorders.. Aust. Prescr..

[23] Willner K., Vasan S., Patel P., Abdijadid S. (2024). Atypical antipsychotic agents.. StatPearls Publishing.

[24] Stocks J.D., Taneja B.K., Baroldi P., Findling R.L. (2012). A phase 2a randomized, parallel group, dose-ranging study of molindone in children with attention-deficit/hyperactivity disorder and persistent, serious conduct problems.. J. Child Adolesc. Psychopharmacol..

[25] Oades R. (2008). Dopamine-serotonin interactions in attention-deficit hyperactivity disorder (ADHD).. Prog. Brain Res..

[26] Pretorius Ε. (2004). Corticosteroids, depression and the role of serotonin.. Rev. Neurosci..

[27] Zhang G., Stackman R.W. (2015). The role of serotonin 5-HT2A receptors in memory and cognition.. Front. Pharmacol..

[28] Albert P.R., Le François B., Vahid-Ansari F. (2019). Genetic, epigenetic and posttranscriptional mechanisms for treatment of major depression: The 5-HT1A receptor gene as a paradigm.. J. Psychiatry Neurosci..

[29] Biederman J., Hammerness P., Doyle R., Joshi G., Aleardi M., Mick E. (2008). Risperidone treatment for ADHD in children and adolescents with bipolar disorder.. Neuropsychiatr. Dis. Treat..

[30] Naguy A. (2016). Low-dose quetiapine complements stimulant response in attention deficit hyperactivity disorder and more.. Ther. Adv. Psychopharmacol..

[31] Madhyastha S., Rao M.S., Renno W.M. (2024). Serotonergic and adrenergic neuroreceptor manipulation ameliorates core symptoms of ADHD through modulating dopaminergic receptors in spontaneously hypertensive rats.. Int. J. Mol. Sci..

[32] Andreollo N.A., Santos E.F., Araújo M.R., Lopes L.R. (2012). Rat’s age versus human’s age: What is the relationship?. Arq. Bras Cir. Dig..

[33] Minabe Y., Hashimoto K., Watanabe K.I., Ashby C.R. (2001). Acute and repeated administration of the selective 5-HT(2A) receptor antagonist M100907 significantly alters the activity of midbrain dopamine neurons: An in vivo electrophysiological study.. Synapse.

[34] Kim P., Choi I.H., Dela Pena I.C., Kim H.J., Kwon K.J., Park J.H., Han S.H., Ryu J.H., Cheong J.H., Shin C.Y. (2012). A simple behavioral paradigm to measure impulsive behavior in an animal model of attention deficit hyperactivity disorder (ADHD) of the spontaneously hypertensive rats.. Biomol. Ther..

[35] Bairy K.L., Madhyastha S., Ashok K.P., Bairy I., Malini S. (2007). Developmental and behavioral consequences of prenatal fluoxetine.. Pharmacology.

[36] Bizot J.C., David S., Trovero F. (2011). Effects of atomoxetine, desipramine, d-amphetamine and methylphenidate on impulsivity in juvenile rats, measured in a T-maze procedure.. Neurosci. Lett..

[37] Natesan S., Reckless G.E., Nobrega J.N., Fletcher P.J., Kapur S. (2006). Dissociation between in vivo occupancy and functional antagonism of dopamine D2 receptors: Comparing aripiprazole to other antipsychotics in animal models.. Neuropsychopharmacology.

[38] Shi Y.X., Han F., Shi Y.X. (2011). Changes in 5-HT1A receptor in the dorsal raphe nucleus in a rat model of post-traumatic stress disorder.. Mol. Med. Rep..

[39] Verdurand M., Chauveau F., Daoust A., Morel A.L., Bonnefoi F., Liger F., Bérod A., Zimmer L. (2016). Differential effects of amyloid-beta 1-40 and 1-42 fibrils on 5-HT 1A serotonin receptors in rat brain.. Neurobiol. Aging.

[40] McDonald A.J., Mascagni F. (2007). Neuronal localization of 5-HT type 2A receptor immunoreactivity in the rat basolateral amygdala.. Neuroscience.

[41] Romero-Fernandez W., Borroto-Escuela D.O., Vargas-Barroso V., Narváez M., Di Palma M., Agnati L.F., Larriva Sahd J., Fuxe K. (2014). Dopamine D1 and D2 receptor immunoreactivities in the arcuate-median eminence complex and their link to the tubero-infundibular dopamine neurons.. Eur. J. Histochem..

[42] Bennett S.A.L., Roberts D.C.S. (2003). Analysis of protein expression in brain tissue by ELISA.. Methods Mol. Med..

[43] Banerjee E., Nandagopal K. (2015). Does serotonin deficit mediate susceptibility to ADHD?. Neurochem. Int..

[44] Nikolaus S., Chao O.Y., Beu M., Henke J., Antke C., Wang A.L., Fazari B., Mamlins E., Huston J.P., Giesel F.L. (2023). The 5-HT1A receptor agonist 8-OH-DPAT modulates motor/exploratory activity, recognition memory and dopamine transporter binding in the dorsal and ventral striatum.. Neurobiol. Learn. Mem.

[45] Anastasio N.C., Stutz S.J., Fink L.H., Swinford-Jackson S.E., Sears R.M., DiLeone R.J., Rice K.C., Moeller F.G., Cunningham K.A. (2015). Serotonin (5-HT) 5-HT2A receptor (5-HT2AR): 5-HT2CR imbalance in medial prefrontal cortex associates with motor impulsivity.. ACS Chem. Neurosci..

[46] Arnsten A.F. (2009). The emerging neurobiology of attention deficit hyperactivity disorder: The key role of the prefrontal association cortex.. J. Pediatr..

[47] Kawahata I., Finkelstein D.I., Fukunaga K. (2024). Dopamine D1-D5 receptors in brain nuclei: Implications for health and disease.. Receptors.

[48] Jenni N.L., Larkin J.D., Floresco S.B. (2017). Prefrontal dopamine D1 and D2 receptors regulate dissociable aspects of decision-making via distinct ventral striatal and amygdalar circuits.. J. Neurosci..

[49] Wingen M., Kuypers K.P.C., Ramaekers J.G. (2007). The role of 5-HT1a and 5-HT2a receptors in attention and motor control: A mechanistic study in healthy volunteers.. Psychopharmacology.

[50] Morici J.F., Ciccia L., Malleret G., Gingrich J.A., Bekinschtein P., Weisstaub N.V. (2015). Serotonin 2a receptor and serotonin 1a receptor interact within the medial prefrontal cortex during recognition memory in mice.. Front. Pharmacol..

[51] Brennan A.R., Arnsten A.F.T. (2008). Neuronal mechanisms underlying attention deficit hyperactivity disorder: The influence of arousal on prefrontal cortical function.. Ann. N Y Acad. Sci..

[52] Ott T., Stein A.M., Nieder A. (2023). Dopamine receptor activation regulates reward expectancy signals during cognitive control in primate prefrontal neurons.. Nat. Commun..

[53] Wulaer B., Kunisawa K., Tanabe M., Yanagawa A., Saito K., Mouri A., Nabeshima T. (2021). Pharmacological blockade of dopamine D1- or D2-receptor in the prefrontal cortex induces attentional impairment in the object-based attention test through different neuronal circuits in mice.. Mol. Brain.

[54] Swanson J.M., Volkow N.D. (2009). Psychopharmacology: Concepts and opinions about the use of stimulant medications.. J. Child Psychol. Psychiatry.

[55] Winstanley C.A., Eagle D.M., Robbins T.W. (2006). Behavioral models of impulsivity in relation to ADHD: Translation between clinical and preclinical studies.. Clin. Psychol. Rev..

[56] Loos M., Pattij T., Janssen M.C.W., Counotte D.S., Schoffelmeer A.N.M., Smit A.B., Spijker S., van Gaalen M.M. (2010). Dopamine receptor D1/D5 gene expression in the medial prefrontal cortex predicts impulsive choice in rats.. Cereb. Cortex.

[57] Dalley J.W., Roiser J.P. (2012). Dopamine, serotonin and impulsivity.. Neuroscience.

[58] Carli M., Baviera M., Invernizzi R.W., Balducci C. (2006). Dissociable contribution of 5-HT1A and 5-HT2A receptors in the medial prefrontal cortex to different aspects of executive control such as impulsivity and compulsive perseveration in rats.. Neuropsychopharmacology.

[59] Pattij T., Schoffelmeer A.N. (2015). Serotonin and inhibitory response control: Focusing on the role of 5-HT(1A) receptors.. Eur. J. Pharmacol..

[60] Hadamitzky M., Feja M., Becker T., Koch M. (2009). Effects of acute systemic administration of serotonin2A/C receptor ligands in a delay-based decision-making task in rats.. Behav. Pharmacol..

[61] Blokland A., Sik A., Lieben C. (2005). Evaluation of DOI, 8-OH-DPAT, eticlopride and amphetamine on impulsive responding in a reaction time task in rats.. Behav. Pharmacol..

[62] Winstanley C.A., Dalley J.W., Theobald D.E.H., Robbins T.W. (2004). Fractionating impulsivity: Contrasting effects of central 5-HT depletion on different measures of impulsive behavior.. Neuropsychopharmacology.

[63] Fletcher P.J., Tampakeras M., Sinyard J., Higgins G.A. (2007). Opposing effects of 5-HT2A and 5-HT2C receptor antagonists in the rat and mouse on premature responding in the five-choice serial reaction time test.. Psychopharmacology.

[64] Wang L.J., Chen C.K., Lin S.K., Chen Y.C., Xu K., Huang M.C. (2018). Cognitive profile of ketamine-dependent patients compared with methamphetamine-dependent patients and healthy controls.. Psychopharmacology.

[65] Moallem N., Ray L.A. (2012). Quetiapine improves response inhibition in alcohol dependent patients: A placebo-controlled pilot study.. Pharmacol. Biochem. Behav..

[66] Pompeiano M., Palacios J.M., Mengod G. (1992). Distribution and cellular localization of mRNA coding for 5-HT1A receptor in the rat brain: Correlation with receptor binding.. J. Neurosci..

[67] Riad M., Garcia S., Watkins K.C., Jodoin N., Doucet, Langlois X., El Mestikawy S., Hamon M., Descarries L. (2000). Somatodendritic localization of 5-HT1A and preterminal axonal localization of 5-HT1B serotonin receptors in adult rat brain.. J. Comp. Neurol..

[68] Doherty M.D., Pickel V.M. (2001). Targeting of serotonin 1a receptors to dopaminergic neurons within the parabrachial subdivision of the ventral tegmental area in rat brain.. J. Comp. Neurol..

[69] Haleem D.J. (2013). Extending therapeutic use of psychostimulants: Focus on serotonin-1A receptor.. Prog. Neuropsychopharmacol. Biol. Psychiatry.

[70] Carta M., Carlsson T., Kirik D., Björklund A. (2007). Dopamine released from 5-HT terminals is the cause of L-DOPA-induced dyskinesia in parkinsonian rats.. Brain.

[71] Hayes D.J., Greenshaw A.J. (2011). 5-HT receptors and reward-related behaviour: A review.. Neurosci. Biobehav. Rev..

[72] Raote I., Bhattacharya A., Panicker M.M., Chattopadhyay A. (2007). Serotonin 2A (5-HT2A) Receptor Function: Ligand-Dependant Mechanisms and Pathways.. Serotonin Receptors in Neurobiology.

[73] Jakab R.L., Goldman-Rakic P.S. (2000). Segregation of serotonin 5-HT2A and 5-HT3 receptors in inhibitory circuits of the primate cerebral cortex.. J. Comp. Neurol..

[74] López-Giménez J.F., Vilaró M.T., Palacios J.M., Mengod G. (2001). Mapping of 5-HT2A receptors and their mRNA in monkey brain: [3H]MDL100,907 autoradiography and in situ hybridization studies.. J. Comp. Neurol..

[75] McMahon L.R., Filip M., Cunningham K.A. (2001). Differential regulation of the mesoaccumbens circuit by serotonin 5-HT2A and 5-HT2C receptors.. J. Neurosci..

[76] Fink K.B., Göthert M. (2007). 5-HT receptor regulation of neurotransmitter release.. Pharmacol. Rev..

[77] Oades R.D. (2002). Dopamine may be ‘hyper’ with respect to noradrenaline metabolism, but ‘hypo’ with respect to serotonin metabolism in children with attention-deficit hyperactivity disorder.. Behav. Brain Res..

[78] Oades R.D. (2007). Role of the serotonin system in ADHD: Treatment implications.. Expert Rev. Neurother..

[79] Lin X., Huang L., Huang H., Ke Z., Chen Y. (2023). Disturbed relationship between glucocorticoid receptor and 5-HT1AR/5-HT2AR in ADHD rats: A correlation study.. Front. Neurosci..

[80] Di Giovanni G., Esposito E., Di Matteo V. (2010). Role of serotonin in central dopamine dysfunction.. CNS Neurosci. Ther..

[81] Hervé D., Pickel V.M., Joh T.H., Beaudet A. (1987). Serotonin axon terminals in the ventral tegmental area of the rat: Fine structure and synaptic input to dopaminergic neurons.. Brain Res..

[82] Perry K.W., Fuller R.W. (1992). Effect of fluoxetine on serotonin and dopamine concentration in microdialysis fluid from rat striatum.. Life. Sci..

[83] Ichikawa J., Meltzer H.Y. (1995). Effect of antidepressants on striatal and accumbens extracellular dopamine levels.. Eur. J. Pharmacol..

[84] Bymaster F.P., Zhang W., Carter P.A., Shaw J., Chernet E., Phebus L., Wong D.T., Perry K.W. (2002). Fluoxetine, but not other selective serotonin uptake inhibitors, increases norepinephrine and dopamine extracellular levels in prefrontal cortex.. Psychopharmacology.

[85] Bortolozzi A., Díaz-Mataix L., Scorza M.C., Celada P., Artigas F. (2005). The activation of 5-HT receptors in prefrontal cortex enhances dopaminergic activity.. J. Neurochem..

[86] Beaulieu J.M. (2012). A role for Akt and glycogen synthase kinase-3 as integrators of dopamine and serotonin neurotransmission in mental health.. J. Psychiatry Neurosci..

[87] de Bartolomeis A., Buonaguro E.F., Iasevoli F. (2013). Serotonin-glutamate and serotonin-dopamine reciprocal interactions as putative molecular targets for novel antipsychotic treatments: From receptor heterodimers to postsynaptic scaffolding and effector proteins.. Psychopharmacology.

[88] Schaus J.M., Bymaster F.P. (1998). Dopaminergic approaches to antipsychotic agents.. Annu. Rep. Med. Chem..

[89] Fuxe K., Borroto-Escuela D.O., Tarakanov A.O., Romero-Fernandez W., Ferraro L., Tanganelli S., Perez-Alea M., Di Palma M., Agnati L.F. (2014). Dopamine D2 heteroreceptor complexes and their receptor-receptor interactions in ventral striatum.. Prog. Brain Res..

[90] Pehek E.A., Nocjar C., Roth B.L., Byrd T.A., Mabrouk O.S. (2006). Evidence for the preferential involvement of 5-HT2A serotonin receptors in stress- and drug-induced dopamine release in the rat medial prefrontal cortex.. Neuropsychopharmacology.

[91] Meltzer H.Y., Massey B.W. (2011). The role of serotonin receptors in the action of atypical antipsychotic drugs.. Curr. Opin. Pharmacol..

[92] Olijslagers J.E., Werkman T.R., McCreary A.C., Siarey R., Kruse C.G., Wadman W.J. (2004). 5-HT2 receptors differentially modulate dopamine-mediated auto-inhibition in A9 and A10 midbrain areas of the rat.. Neuropharmacology.

[93] Konradi C., Heckers S. (2001). Antipsychotic drugs and neuroplasticity: Insights into the treatment and neurobiology of schizophrenia.. Biol. Psychiatry.

[94] Volkow N.D., Wang G.J., Fowler J.S., Logan J., Angrist B., Hitzemann R., Lieberman J., Pappas N. (1997). Effects of methylphenidate on regional brain glucose metabolism in humans: Relationship to dopamine D2 receptors.. Am. J. Psychiatry.

[95] Willner K., Vasan S., Patel P., Abdijadid S. (2025). Atypical Antipsychotic Agents..

[96] Yanofski J. (2010). The dopamine dilemma: Using stimulants and antipsychotics concurrently.. Psychiatry.

[97] OʼSullivan S.S., Evans A.H., Lees A.J. (2009). Dopamine dysregulation syndrome: An overview of its epidemiology, mechanisms and management.. CNS Drugs.

[98] Tsai L.H., Lin J.W., Lee M.C. (2022). Social communication disorder in an adolescent with ADHD and Tourette’s disorder treated with aripiprazole.. Psychiatr. Danub.

[99] Pan P.Y., Fu A.T., Yeh C.B. (2018). Aripiprazole/methylphenidate combination in children and adolescents with disruptive mood dysregulation disorder and attention-deficit/hyperactivity disorder: An open-label study.. J. Child Adolesc. Psychopharmacol..

